# Application of Whole Genome Sequencing and Pan-Family Multi-Locus Sequence Analysis to Characterize Relationships Within the Family *Brucellaceae*

**DOI:** 10.3389/fmicb.2020.01329

**Published:** 2020-07-14

**Authors:** Roland T. Ashford, Jakub Muchowski, Mark Koylass, Holger C. Scholz, Adrian M. Whatmore

**Affiliations:** ^1^Department of Bacteriology, Animal and Plant Health Agency, Weybridge, United Kingdom; ^2^Department of Bacteriology and Toxinology, Bundeswehr Institute of Microbiology, Munich, Germany

**Keywords:** *Brucella*, *Brucellaceae*, multi-locus locus sequence analysis, pan-family, phylogeny, *Ochrobactrum*

## Abstract

The bacterial family *Brucellaceae* is currently composed of seven genera, including species of the genus *Brucella*, a number of which are significant veterinary and zoonotic pathogens. The bacteriological identification of pathogenic *Brucella* spp. may be hindered by their close phenotypic similarity to other members of the *Brucellaceae*, particularly of the genus *Ochrobactrum*. Additionally, a number of novel atypical *Brucella* taxa have recently been identified, which exhibit greater genetic diversity than observed within the previously described species, and which share genomic features with organisms outside of the genus. Furthermore, previous work has indicated that the genus *Ochrobactrum* is polyphyletic, raising further questions regarding the relationship between the genus *Brucella* and wider *Brucellaceae*. We have applied whole genome sequencing (WGS) and pan-family multi-locus sequence analysis (MLSA) approaches to a comprehensive panel of *Brucellaceae* type strains, in order to characterize relationships within the family. Phylogenies based on WGS core genome alignments were able to resolve phylogenetic relationships of 31 non-*Brucella* spp. type strains from within the family, alongside type strains of twelve *Brucella* species. A phylogeny based on concatenated pan-family MLSA data was largely consistent with WGS based analyses. Notably, recently described atypical *Brucella* isolates were consistently placed in a single clade with existing species, clearly distinct from all members of the genus *Ochrobactrum* and wider family. Both WGS and MLSA methods closely grouped *Brucella* spp. with a sub-set of *Ochrobactrum* species. However, results also confirmed that the genus *Ochrobactrum* is polyphyletic, with seven species forming a separate grouping. The pan-family MLSA scheme was subsequently applied to a panel of 50 field strains of the family *Brucellaceae*, isolated from a wide variety of sources. This analysis confirmed the utility of the pan-*Brucellaceae* MLSA scheme in placing field isolates in relation to recognized type strains. However, a significant number of these isolates did not cluster with currently identified type strains, suggesting the existence of additional taxonomic diversity within some members of the *Brucellaceae*. The WGS and pan-family MLSA approaches applied here provide valuable tools for resolving the identity and phylogenetic relationships of isolates from an expanding bacterial family containing a number of important pathogens.

## Introduction

The bacterial family *Brucellaceae* (class *Alphaproteobacteria*, order *Rhizobiales*) is currently comprised of seven genera; *Brucella*, *Daeguia, Falsochrobactrum, Mycoplana, Ochrobactrum, Paenochrobactrum*, and *Pseudochrobactrum* (Kämpfer and Glaeser, 2019). The family contains species with a wide range of habitat or host preferences, encompassing obligate intracellular pathogens of animals (e.g., *Brucella melitensis*), opportunistic pathogens often associated with nosocomial infections (e.g., *Ochrobactrum anthropi*), plant associated pathogens and symbionts (e.g., *O. lupini*) and organisms isolated from the natural and anthropogenic environment (e.g., *Paenochrobactrum glaciei* and *Pseudochrobactrum lubricantis*, respectively).

The type genus *Brucella* (Scholz et al., 2018) contains the causative agents of brucellosis, which remains one of the most important zoonotic diseases globally, with more than 500,000 new cases reported each year (Pappas et al., 2006). For several decades the genus *Brucella* was described as consisting of six “classical” species (*B. abortus*, *B. melitensis*, *B. ovis*, *B. suis*, *B. canis*, and *B. neotomae*), with well characterized mammalian host preferences (Corbel, 2006). Subsequently, additional species have been described, with the genus expanding to include *B. pinnipedialis* and *B. ceti* isolated from marine mammals (Foster et al., 2007), *B. microti* from voles (Scholz et al., 2008b), *B. inopinata* isolated from a human infection (Scholz et al., 2010), *B. papionis* from baboons (Whatmore et al., 2014) and *B. vulpis*, isolated from foxes (Scholz et al., 2016a). Additionally, a number of novel atypical strains from human and other mammalian hosts have been described, but await formal classification (Tiller et al., 2010a, b; Guzmán-Verri et al., 2019). These species and strains reflect an ongoing expansion of the known host range and genetic diversity of the genus. In particular, *B. microti*, *B. inopinata* and *B. vulpis* have been described as “atypical” *Brucella* species, exhibiting either atypical phenotypic traits (*B. microti*), or greater genetic diversity (*B. inopinata* and *B. vulpis*).

In addition to the novel atypical *Brucella* species and strains identified from mammalian hosts there is an expanding body of literature describing the isolation of *Brucella* sp. organisms from poikilothermic hosts, namely amphibians (Eisenberg et al., 2012; Whatmore et al., 2015; Scholz et al., 2016b; Soler-Lloréns et al., 2016) and cartilaginous fish (Eisenberg et al., 2017). In many cases, these isolates have initially been misidentified as *Ochrobactrum anthropi* on the basis of automated phenotyping or mass spectrometry (Eisenberg et al., 2017). However, further genetic investigation has identified the isolates as representing distinct *Brucella* lineages (Scholz et al., 2016b). Such isolates fall outside of the core *Brucella*, exhibiting greatest similarity to previously described atypical isolates from mammals (*B. inopinata* and *B. vulpis*). Analyses based on whole genome sequencing (WGS) have indicated that, whilst such amphibian isolates maintain a high degree of genetic homology with core *Brucella* species, they exhibit a degree of horizontal gene transfer, with the incorporation of genomic regions exhibiting sequence identity to soil living or facultatively pathogenic *Alphaproteobacteria*, most notably from the genus *Ochrobactrum* (Scholz et al., 2016b; Al Dahouk et al., 2017).

The genus *Ochrobactrum*, is the largest within the family *Brucellaceae*, and has expanded significantly in the past decade (Kämpfer et al., 2018). The genus currently consists of 18 validly published species, plus a number of others awaiting valid publication. It has previously been noted that the genus *Ochrobactrum* appears to be polyphyletic (Kämpfer et al., 2013). Furthermore, studies based on sequencing of single locus targets (most commonly 16S ribosomal RNA or *recA*), have generated conflicting results on phylogenetic relationships within the family.

These issues highlight the well described problems of inferring phylogenetic relationships from single genetic loci, including lack of resolution, stochastic variation and the influence of recombination or horizontal gene transfer (Gevers et al., 2005). Previously, few studies had attempted to apply a multi-locus analysis approach to interrogate phylogenetic relationships within the family *Brucellaceae*. Aujoulat et al. (2014) applied an existing multi-locus sequence typing scheme for *Ochrobactrum anthropi* (Romano et al., 2009) to a wider panel of type strains from 14 *Ochrobactrum* species and two *Brucella* species. These authors again identified the genus *Ochrobactrum* as polyphyletic, with a robust clade containing *O. anthropi* and *O. intermedium* and five other species grouping distinctly from a less well supported clade containing the remaining *Ochrobactrum* type strains and the two *Brucella* species included. However, this analysis did not incorporate the full diversity of either *Ochrobactrum* or *Brucella*, or the remaining five genera which make up the family.

More recently, a number of studies have combined multi-locus sequencing and whole genome sequencing (WGS) based approaches to address specific taxonomic issues within the genus *Ochrobactrum*. Gazolla Volpiano et al. (2019) for example, identified *O. lupini* as a heterotypic synonym of *O. anthropi*. Similarly, Krzyzanowska et al. (2019) used a combination of these approaches to investigate the relationship of a proposed novel *Ochrobactrum* species (*O. quorumnocens*) to previously described members of the genus.

Very recently, Leclercq et al. (2019) used WGS data to investigate relationships within the family *Brucellaceae*, as well as its position within the wider *Rhizobiales*. These authors confirmed the genus *Brucella* to be a monophyletic grouping within the genus *Ochrobactrum*, and that *Ochrobactrum* spp. divide into two distinct clades. However, Leclercq et al. (2019) did not incorporate type strains reflecting the full taxonomic diversity of either *Ochrobactrum* or *Brucella*. Additionally, the remaining genera within the *Brucellaceae* were either under-represented (*Pseudochrobactrum* and *Mycoplana*) or absent (*Paenochrobactrum* and *Daeguia*).

The continuing expansion of the recognized genetic diversity of the genus *Brucella*, and the uncertainty regarding phylogenetic relationships amongst members of related genera, highlight the need for a comprehensive multi-gene phylogeny of the *Brucellaceae*. Here, we describe the parallel application of WGS and multi-locus sequence analysis (MLSA) approaches to investigate phylogenetic relationships within the family. The pan-family MLSA method developed incorporates loci identical those used in existing schemes for *Brucella* species (Whatmore et al., 2007, 2016) thereby permitting the comparison of data generated under both schemes. Subsequently, we have applied the pan-family MLSA scheme to panels of field isolates, to assess its utility in establishing relationships with extant species.

## Materials and Methods

### Type Strains

The taxonomic status of species within the *Brucellaceae* was retrieved via the online resource *List of Prokaryotic Names with Standing in Nomenclature* (Euzeby, 1997; Parte, 2014 accessed 14/11/2019). The type strains of all *Brucella* species were available from the collection of the APHA *Brucella* Reference Laboratory. The type strains of other species identified as belonging to the family *Brucellaceae* were purchased from established culture collections (Table 1). Strains were grown on solid media at optimal conditions as described by the supplier. A single pure colony from each agar plate was transferred to 100 μl of molecular grade water and cells were lysed by heating at 100°C for 10 min. Type strain 16S rRNA sequences were retrieved from the National Centre for Biotechnology Information (NCBI) database.

**TABLE 1:** Type strains of species belonging to the *Brucellaceae*, used for whole genome sequence analysis and pan-family multi-locus sequence analysis (genus type species shown in bold).

**Genus**	**Species**	**Type strain**	**Valid publication**	**WGS data**
*Brucella*	*B. abortus*	NCTC 10093^T^	Meyer and Shaw (1920)	GCA_000739315.1
	*B. canis*	NCTC 10854^T^	Carmichael and Bruner (1968)	GCF_000018525.1
	*B. ceti*	NCTC 12891^T^	Foster et al. (2007)	GCF_000662035.2
	*B. inopinata*	BO1^T^	Scholz et al. (2010)	GCF_000182725.1
	***B. melitensis***	**NCTC 10094^T^**	**Meyer and Shaw (1920)**	GCF_000007125.1
	*B. microti*	CCM 4915^T^	Scholz et al. (2008b)	GCF_000022745.1
	*B. neotomae*	NCTC 10084^T^	Stoenner and Lackman (1957)	GCF_900446125.1
	*B. ovis*	NCTC 10512^T^	Buddle (1956)	GCF_900446135.1
	*B. papionis*	NCTC 13660^T^	Whatmore et al. (2014)	SRR4038994
	*B. pinnipedialis*	NCTC 12890^T^	Foster et al. (2007)	GCF_000221005.1
	*B. suis*	NCTC 10316^T^	Huddleson (1929)	GCF_000007505.1
	*B. vulpis*	DSM 101715^T^	Scholz et al. (2016a)	GCA_900000005.1
*Daeguia*	***D. caeni***	**CCUG 54520^T^**	**Yoon et al. (2008)**	This study*
*Falsochrobactrum*	***F. ovis***	**LMG 27356^T^**	**Kämpfer et al. (2013)**	This study
	*F. shanghaiense*	HN4^T^	Sun et al. (2019)	GCF_003149535.1
*Mycoplana*	***M. dimorpha***	**DSM 7138^T^**	**Gray and Thornton (1928)**	This study
	*M. ramosa*	DSM 7292^T^	Urakami et al. (1990)	This study*
*Ochrobactrum*	***O. anthropi***	**LMG 3331^T^**	**Holmes et al. (1988)**	GCF_000017405.1
	*O. ciceri*	DSM 22292^T^	Imran et al. (2010)	This study
	*O. cytisi*	DSM 19778^T^	Zurdo-Pińeiro et al. (2007)	This study
	*O. daejeonense*	JCM 16234^T^	Woo et al. (2011)	This study
	*O. endophyticum*	DSM 29930^T^	Li et al. (2016)	This study*
	*O. gallinifaecis*	DSM 15295^T^	Kämpfer et al. (2003)	GCF_006476605.1
	*O. grignonense*	LMG 18954^T^	Lebuhn et al. (2000)	This study
	*O. haematophilum*	CIP 109452^T^	Kämpfer et al. (2007a)	This study
	*O. intermedium*	LMG 3301^T^	Velasco et al. (1998)	GCF_000182645.1
	*O. lupini* ^*a*^	DSM 16930^T^	Trujillo et al. (2005)	This study
	*O. oryzae*	DSM 17471^T^	Tripathi et al. (2006)	This study
	*O. pecoris*	CCUG 60088^T^	Kämpfer et al. (2011)	GCF_006376675.1
	*O. pituitosum*	DSM 22207^T^	Huber et al. (2010)	This study
	*O. pseudintermedium*	DSM 17490^T^	Teyssier et al. (2007)	GCF_008932435.1
	*O. pseudogrignonense*	CIP 109451^T^	Kämpfer et al. (2007a)	This study
	*O. quorumnocens*^*b*^	LMG 30544^T^	Krzyzanowska et al. (2019)	GCF_002278035.1
	*O. rhizosphaerae*	DSM 19824^T^	Kämpfer et al. (2008)	This study
	*O. thiophenivorans*	DSM 7216^T^	Kämpfer et al. (2008)	This study
	*O. tritici*	LMG 18957^T^	Lebuhn et al. (2000)	GCF_008932295.1
*Paenochrobactrum*	***P. gallinarii***	**CCUG 57736^T^**	**Kämpfer et al. (2010)**	This study*
	*P. glaciei*	JCM 15115^T^	Romanenko et al. (2008); Kämpfer et al. (2010)	This study*
	*P. pullorum*	LMG 28095^T^	Kämpfer et al. (2014)	This study*
*Pseudochrobactrum*	***P. asaccharolyticum***	**CCUG 46016^T^**	**Kämpfer et al. (2006)**	This study
	*P. kiredjianiae*	DSM 19762^T^	Kämpfer et al. (2007b)	This study*
	*P. lubricantis*	CCUG 56963^T^	Kämpfer et al. (2009)	This study*
	*P. saccharolyticum*	CCUG 33852^T^	Kämpfer et al. (2006)	This study

*Strains marked with an asterisk (^∗^) were sequenced for the first time for this study. ^*a*^*O. lupini* identified as a heterotypic synonym of *O. anthropi*; ^*b*^*O. quorumnocens* not yet validly published.*

### Additional Atypical *Brucella* sp. Strains

In addition to *Brucellaceae* type strains, atypical *Brucella* sp. from both mammalian and cold-blooded hosts were included in phylogenetic analyses (Table 2). These included previously described atypical isolates from a human patient (BO2: Tiller et al., 2010b) and from Australian rodents (Tiller et al., 2010a). These latter strains (referred to here as 83/13 and 83/4) were originally isolated as part of the same cohort as the Australian rodent strain NF2653 (Cook et al., 1966). Additionally, a number of more recently described atypical isolates from amphibians were analyzed. Isolate UK8/14 was recovered from a White’s tree frog (Whatmore et al., 2015). Six African bullfrog strains from the 21 previously analyzed by Scholz et al. (2016b) were also selected, on the basis of representing all sequence types from this panel in the existing *Brucella* MLSA scheme (09RB8471: ST63; 09RB8910: ST64; 09RB8913; ST65; 10RB9205: ST66; 10RB9215: ST67 and 10RB9213: ST68 (Al Dahouk et al., 2017).

**TABLE 2:** Atypical *Brucella* sp. strains used for whole genome sequence analysis and pan-family multi-locus sequence analysis.

**Strain ID**	**Source**	**Reference**	**Accession number**
BO2	Human lung biopsy	Tiller et al. (2010b)	GCA_000177135.1
83/13	Australian rodent	–	GCA_000157875.1
83/4	Australian rodent	–	–
NF2653	Australian rodent	Wattam et al. (2012)	GCA_000177155.1
UK8/14	Whites tree frog (*Litoria caerulea*)	Whatmore et al. (2015)	–
09RB8471	African bull frog (*Pyxicephalus edulis*)	Al Dahouk et al. (2017)	GCA_001971625.1
09RB8910	African bull frog (*Pyxicephalus edulis*)	Al Dahouk et al. (2017)	GCA_001971805.1
09RB8913	African bull frog (*Pyxicephalus edulis*)	Al Dahouk et al. (2017)	GCA_009664925.1
10RB9205	African bull frog (*Pyxicephalus edulis*)	Al Dahouk et al. (2017)	-
10RB9213	African bull frog (*Pyxicephalus edulis*)	Al Dahouk et al. (2017)	GCA_009664915.1
10RB9215	African bull frog (*Pyxicephalus edulis*)	Al Dahouk et al. (2017)	GCA_900092405.1
141012304	Ribbontail ray (*Taeniura lymma*)	Eisenberg et al. (2017)	GCA_900095155.1
B13-0095	Pac-Man frog (*Ceratophrys ornata*)	Soler-Lloréns et al. (2016)	GCA_001742815.1

### Field Strains

The performance of the MLSA scheme was further evaluated using field isolates provided by the Bundeswehr Institute of Microbiology (Munich, Germany) and obtained by the Animal and Plant Health Agency (Weybridge, United Kingdom) via brucellosis surveillance activities. In the former case, these represented a diverse panel of *Ochrobactrum* spp. from a variety of sources and geographical locations, confirmed as *Ochrobactrum* sp. by both 16S rRNA and *recA* sequencing (see Scholz et al., 2008a for full details). A panel of 37 isolates was examined (Table 3), representing major clades identified by *recA* sequence analysis (Scholz et al., 2008a). DNA was prepared as previously described (Scholz et al., 2008a). In the case of Animal and Plant Health Agency (APHA) isolates (*n* = 13), these were submitted as suspect *Brucella* sp. of veterinary origin but subsequently identified as non-*Brucella* members of the *Brucellaceae* by 16S rRNA sequencing (Wragg et al., 2014) (Table 3). Pure cultures were stored frozen in either Luria Broth with 15% glycerol or using proprietary MicroBank tubes (ProLab Diagnostics, United Kingdom). Frozen strains were revived by plating onto sheep blood and/or nutrient agar and incubating at 28°C for 24–48 h. Crude thermo-lysates from single colonies were prepared as described above.

**TABLE 3:** Field strains used for evaluation of the pan-*Brucellaceae* multi-locus sequence analysis scheme.

**Strain ID^*a*^**	**Source**	**Geographic origin**	**Identity^*b*^**
CCM 7036 (D)	*Phlebotomus duboscqi*	Czechia	*O. intermedium*
CCUG 1047 (D)	Dextran	Sweden	*O. anthropi*
CCUG 12860 (D)	Human blood	Sweden	*O. anthropi*
CCUG 16508 (D)	n/a	France	*O. anthropi*
CCUG 17894 (D)	Contaminant	Sweden	*O. anthropi*
CCUG 1821 (D)	Cattle dip trays	Australia	*O. tritici*
CCUG 29689 (D)	Human blood	Sweden	*O. tritici*
CCUG 32009 (D)	Paint	Sweden	*O. anthropi*
CCUG 33786 (D)	Human blood	Sweden	*O. anthropi*
CCUG 34735 (D)	Water	Sweden	*O. pseudintermedium*
CCUG 39736 (D)	Human blood	Sweden	*O. intermedium*
CCUG 43465 (D)	Paper mill	Sweden	*O. pseudintermedium*
CCUG 43892 (D)	Human Ear	Norway	*O. pseudogrignonense*
CCUG 7349 (D)	n/a	n/a	*O. anthropi*
CLM 26 (D)	Soil	Germany	*O. anthropi*
CLM 6 (D)	Soil	Germany	*O. anthropi*
DSM 20150 (D)	Urine of leech	Germany	*O. anthropi*
LMG 2134 (D)	Cattle dip trays	Australia	*O. tritici*
LMG 2136 (D)	Sewage plant	Sweden	*O. anthropi*
LMG 3298 (D)	Blood	United States	*O. anthropi*
LMG 3306 (D)	Soil	France	*O. intermedium*
LMG 35 (D)	Human sputum	Denmark	*O. anthropi*
LMG 394 (D)	Exudate	United States	*O. anthropi*
LMG 401 (D)	Cervix	United States	*O. tritici*
LMG 5425 (D)	Urine	United Kingdom	*O. intermedium*
LMG 5426 (D)	Urine	United Kingdom	*O. intermedium*
LMG 5442 (D)	Throat	United States	*O. anthropi*
LMG 5444 (D)	Human	United States	*O. anthropi*
LMG 5446 (D)	Bladder	United States	*O. intermedium*
LMG 379 (D)	Ear	United States	*O. intermedium*
RR (D)	Industrial environment	Austria	*O. intermedium*
OgA9c (D)	Soil	France	*O. grignonense*
TA13 (D)	Industrial environment	Austria	*O. intermedium*
TA93 (D)	Industrial environment	Austria	*O. tritici*
TD30 (D)	Industrial environment	Austria	*O. intermedium*
WS1830 (D)	Wheat rhizoplane	Germany	*O. tritici*
WS1846 (D)	*Oryza sativa*	India	*O. oryzae*
R5/07 (UK)	*Bos taurus*	United Kingdom	*Pseudochrobactrum* sp.
R16/08-3UT (UK)	*Capra hircus*	Italy	*Pseudochrobactrum* sp.
R17/08-1UD (UK)	*Bubalus bubalis*	Italy	*Pseudochrobactrum* sp.
R22/05 (UK)	*Bos taurus*	United Kingdom	*Pseudochrobactrum* sp.
R24/05 (UK)	*Bos taurus*	United Kingdom	*Pseudochrobactrum* sp.
UK2/05 (UK)	*Bos taurus*	United Kingdom	*Pseudochrobactrum* sp.
UK3/11-1 (UK)	*Ovis aries*	United Kingdom	*Paenochrobactrum* sp.
UK5/07 (UK)	*Bos taurus*	United Kingdom	*Pseudochrobactrum* sp.
UK14/09 (UK)	*Bos taurus*	United Kingdom	*Pseudochrobactrum* sp.
UK20/09 (UK)	*Bos taurus*	United Kingdom	*Pseudochrobactrum* sp.
UK34/07-2 (UK)	*Bos taurus*	United Kingdom	*Pseudochrobactrum* sp.
VLA10/07-E (UK)	*Phocoena phocoena*	United Kingdom	*Pseudochrobactrum* sp.
VLA07/50 (UK)	*Phocoena phocoena*	United Kingdom	*Ochrobactrum* sp.

*^*a*^ Strains denoted (D) provided by Bundeswehr Institute of Microbiology (Munich, Germany); strains denoted (UK) obtained by the Animal and Plant Health Agency (Weybridge, United Kingdom. ^*b*^ Identity based on published data (strains denoted D: see Scholz et al., 2008a) or analysis of 16S rRNA sequence (strains denoted UK).*

### Multi-Locus Sequence Analysis (MLSA)

Genomic targets for the pan-*Brucellaceae* MLSA scheme were selected from a larger panel of 21 loci used in an existing scheme for the *Brucella* genus (Whatmore et al., 2016). Alignments of sequences retrieved from NCBI were constructed for all 21 *Brucella* genus MLSA loci using CLUSTALW, implemented in the Lasergene software suite (version 12, DNASTAR Inc., WI, United States), for processing by the degenerate primer design program HYDEN (Linhart and Shamir, 2007) using default settings (25 bp in length, up to 1 mis-match in the 5′ and 3′ region allowed). Further iterations of primers were generated as alternatives, by modifying these default settings (primer length, number of mis-matches allowed and the level of degeneracy allowed across the primer).

Thermo-lysates from *Brucellaceae* type strains (generated as described above) were used to evaluate MLSA targets and optimize PCR conditions, to identify a panel of loci which amplified reliably using a standard thermal profile and reaction conditions. Optimal reaction conditions were identified as 5.0 μl of Roche 10x PCR buffer, 200 μM final concentration of dNTPs, 1.25 units of FastStart *Taq* polymerase, 2 mM of MgCl_2_, 0.4 μM of forward and reverse primers and 1 μl of template DNA (thermo-lysate), in a total reaction volume of 50 μl. Amplification was performed using an Eppendorf MasterCyclerPro (Eppendorf, Germany), with the following thermal cycle: 94°C for 5 min. followed by 35 cycles of 94°C for 1 min., 55°C for 1 min. and 72°C for 1 min. and a final extension step of 72°C for 7 min. Products were separated by agarose gel electrophoresis to confirm that only a single product of the expected size was present. PCR products were then purified by passage through QIAquick PCR purification columns (Qiagen, United Kingdom) and sequenced in both directions using the same forward and reverse primers as used for initial PCR amplification. The Big Dye Terminator Cycle Sequencing Kit (version 3.1, Applied Biosystems, Carlsbad, CA, United States) was used according to manufacturer’s instructions, with capillary electrophoresis on an ABI 3130xl DNA Analyzer (Applied Biosystems, Carlsbad, CA, United States). In a small number of cases an amplified product of the correct size was extracted from an agarose gel following electrophoresis and purified using the MinElute Gel Extraction Kit (Qiagen, United Kingdom), according to manufacturer’s instructions. Purified PCR products were then sequenced as described.

Sanger sequence data were manipulated using the Lasergene software suite (version 12, DNASTAR Inc., WI, United States), to produce a trimmed consensus sequence file for each strain and locus. Consensus sequences were used to perform phylogenetic analyses, for both individual loci and concatenated sequences (in the following order: *gpd*, *dnaK*, *trpE*, *csdB*, *leuA*, and *acnA*). The Lasergene MegAlign module was used to estimate percentage similarity between sequences. Other summary statistics were calculated using MEGA5 (version 5.2; Tamura et al., 2011). Three type strains were not available when MLSA work was undertaken (*O. endophyticum* DSM 29930^T^; *O. quorumnocens* LMG 30544^T^; *F. shanghaiense* HN4^T^), and therefore MLSA sequences were retrieved from WGS data as described below. Similarly, MLSA data for three atypical *Brucella* sp. isolates (141012304, B13-0095 and NF2653) were extracted from WGS data available on NCBI. Phylogenetic analyses for MLSA data were performed using MEGA5. Maximum likelihood trees were constructed using a general time reversible substitution model (gamma distribution plus invariant sites), with percentage bootstrap confidence levels of internal branches calculated from 1000 re-samplings of the original data. Sequence data from *Rhizobium etli* CFN42^T^ (*Rhizobiales*; *Rhizobiaceae*; assembly accession GCF_000092045.1) were included and defined as an out-group in all analyses.

### Whole Genome Sequencing (WGS)

Whole genome sequencing data from type strains were either retrieved from publically available databases (NCBI), or generated for this study (Table 1). For this purpose DNA was extracted from crude thermo-lysates, using the Qiagen DNEasy Blood and Tissue Kit (Qiagen, Manchester, United Kingdom). Library preparation was performed using the Nextera XT Library Preparation Kit (Illumina Inc., San Diego, CA, United States) according to the manufacturer’s protocol. Libraries were sequenced on the Illumina MiSeq platform, using the MiSeq v2 Reagent Kit (Illumina Inc., San Diego, CA, United States), producing 150 bp paired-end reads.

Raw sequence data were processed using the Nullarbor pipeline (version 2.0^1^), which integrates a number of previously published bioinformatic tools. Briefly, quality assessment and trimming of raw read data was performed using Trimmomatic (version 0.39; Bolger et al., 2014). *De novo* assembly of raw reads was performed using the SPAdes assembler (version 3.13.1; Bankevich et al., 2012). *De novo* assembled genomes were then annotated using Prokka (version 1.13.1; Seemann, 2014). Genomes retrieved from publically available databases (either as finished genomes or whole genome shotgun assembly contigs) were likewise annotated using Prokka. Where an assembled genome was not available via NCBI then short-read data were downloaded from the NCBI SRA database and assembled as described. Genomic average nucleotide identity (ANI) estimates were calculated from fasta formatted genome assemblies, using FastANI (version 1.3; Jain et al., 2018).

In order to generate pan-genomes from WGS data, Roary (version 3.13.0; Page et al., 2015) was used, with a minimum sequence identity of 70% and gene presence in ≥99% of isolates to be identified as a core gene. Core genome sequence alignments were generated using MAFFT (Katoh et al., 2002). Core genome alignments were used to produce maximum likelihood phylogenies with RAxML-NG (Kozlov et al., 2019) applying a general time reversible model (gamma distribution plus invariant sites), with 100 bootstraps. The resulting phylogenies were viewed and further annotated using MEGA5. *Rhizobium etli* CFN42^T^ (*Rhizobiales*; *Rhizobiaceae*; assembly accession GCF_000092045.1) was included and defined as an out-group. Additional outgroup strains were incorporated in some analyses to investigate the placement of *Mycoplana* spp. (*Agrobacterium tumefaciens* Ach5: GCF_000971565.1; *Aquamicrobium aerolatum* DSM 21857^T^: GCF_900113935.1; *Ensifer adhaerens* ATCC 33212^T^: GCF_000697965.2; *Ensifer sojae* DSM 26426^T^: GCF_000261485.1; *Mesorhizobium ciceri*: WSM 1271: GCF_000185905.1; *Mesorhizobium loti* NZP 2037^T^: GCF_001676765.1; *Rhizobium etli* CFN42^T^: GCF_000092045.1; *Sinorhizobium meliloti* DSM 30135^T^: GCF_000006965.1).

## Results

### WGS Core Genome Analyses and Selection of MLSA Loci

Summary statistics for *de novo* genome assemblies are provided in Supplementary Table S1. Of those type strain genomes sequenced for this study (see Table 1) the genome size ranged from 3.2 to 5.8 Mb, corresponding to between 3030 and 5542 annotated coding sequences (Supplementary Table S1). The mean GC content of these genomes ranged from 48.6% to 63.5%. Pan genome analysis of annotated assemblies identified a core genome of 450 genes amongst the 43 type strains included in the analysis (plus *R. etli* CFN42^T^). The concatenated core genome alignment of these genes comprised 449,688 bp. Inclusion of additional outgroup strains reduced the core genome to 410 genes (419,690 bp), whilst inclusion of atypical *Brucella* strains resulted in a core genome of 400 genes (395,037 bp).

Six loci were identified which amplified reliably in all type species of the *Brucellaceae*, using the standard thermal profile and reaction conditions described, and were therefore incorporated into the pan-family MLSA scheme (Supplementary Table S2). All loci represent informative housekeeping genes of the type conventionally used in MLSA. Mean GC content in the six loci ranged from 54.5% to 59.4%, with an average of 57.0% across all loci. The proportion of polymorphic sites in each of the six loci, across all *Brucellaceae* type species, ranged from 42.34% (*dnaK*) to 53.50% (*trpE*), with an average of 48.37%. Nucleotide diversity (*π*) in the six loci ranged from 0.1256 (*dnaK*) to 0.1975 (*trpE*), whilst nucleotide diversity across the entire 3,004 bp concatenated sequence was 0.1532 (Supplementary Table S2).

### Sequence Diversity Indices From WGS and MLSA

The level of genomic ANI and MLSA nucleotide identity observed within and between genera of the *Brucellaceae* varied considerably (Table 4 and Supplementary Table S3). As anticipated, the highest intra-generic ANI values were observed within the *Brucella* (average 99.1%) whilst the lowest average ANI value was seen amongst *Falsochrobactrum* (average 77.9%). The largest intra-generic range (highest to lowest) in ANI values was observed within the *Ochrobactrum* genus (75.4–98.5%). Between genera, the highest ANI was observed between *Brucella* and *Ochrobactrum* (80.9%), whilst the lowest was between *Mycoplana* and *Paenochrobactrum* or *Pseudochrobactrum* (both 74.3%). The same pattern was observed in nucleotide identity estimates derived from MLSA data (Table 4). Again, nucleotide identity within the genus *Brucella* was very high (average 99.3%). Average nucleotide identity within the other genera of the *Brucellaceae* ranged from 79.4% (*Falsochrobactrum*) to 93.7% (*Pseudochrobactrum*). The largest intra-generic range (highest to lowest) in nucleotide identity was observed within the *Ochrobactrum* (78.2–99.7%). Between genera, the highest level of average nucleotide identity was observed between *Brucella* and *Ochrobactrum* (86.9%), whilst the lowest was between *Mycoplana* and *Paenochrobactrum* (70.7%).

**TABLE 4:** Intra-generic (bold) and inter-generic similarity of seven genera of the family *Brucellaceae*, measured by genomic ANI values from WGS data (bottom left matrix) and nucleotide identity from concatenated MLSA data (upper right matrix).

**Genus (*n* species)**	***Brucella* (*n* = 12)**	***Daeguia* (*n* = 1)**	***Falsochrobactrum* (*n* = 2)**	***Mycoplana* (*n* = 2)**	***Ochrobactrum* (*n* = 19)**	***Paenochrobactrum* (*n* = 3)**	***Pseudochrobactrum* (*n* = 4)**
*Brucella*	**99.3 (98–100)**	86.0 (85.8–86.1)	82.0 (79.9–84.1)	77.7 (77.4–77.9)	86.9 (82.2–88.9)	76.8 (76.7–77.2)	78.4 (78.0–78.8)
	**99.1 (97.5–99.9)**						
*Daeguia*	79.4 (79.4–79.5)	**NA**	81.3 (80.3–82.3)	78.4 (78.3–78.5)	85.2 (81.3–86.8)	78.9 (78.5–79.4)	79.6 (79.0–80.1)
		**NA**					
*Falsochrobactrum*	78.5 (76.7–80.4)	77.6 (76.5–78.7)	**79.4 (79.4–79.4)**	74.7 (71.8–78.2)	81.8 (76.2–84.8)	76.8 (75.1–78.7)	77.1 (75.8–78.0)
			**77.9 (77.9–77.9)**				
*Mycoplana*	75.5 (75.4–75.6)	75.3 (75.3–75.3)	74.8 (74.2–75.4)	**93.5 (93.5–93.5)**	77.4 (73.7–81.2)	70.7 (70.2–71.0)	72.6 (71.3–73.2)
				**84.8 (84.8–84.8)**			
*Ochrobactrum*	80.9 (77.4–82.9)	78.2 (76.7–79.3)	77.9 (74.8–80.5)	75.1 (74.4–76.4)	**88.0 (78.2–99.7)**	77.3 (74.3–78.6)	78.6 (75.4–80.2)
					**82.2 (75.4–98.5)**		
*Paenochrobactrum*	76.0 (75.8–76.2)	76.1 (76.0–76.2)	75.8 (75.7–76.0)	74.3 (74.2–74.3)	75.8 (74.8–76.4)	**92.3 (90.4–95.6)**	79.6 (79.2–80.5)
						**88.4 (86.3–92.3)**	
*Pseudochrobactrum*	75.9 (75.6–76.2)	75.9 (75.6–76.2)	75.6 (75.3–76.0)	74.3 (74.2–74.4)	75.9 (74.7–76.9)	77.1 (76.8–77.5)	**93.7 (92.1–97.5)**
							**88.7 (85.8–97.1)**

*Values are expressed as the average for each group (with range). NA, not available where genus represented by a single species.*

**FIGURE 1:**
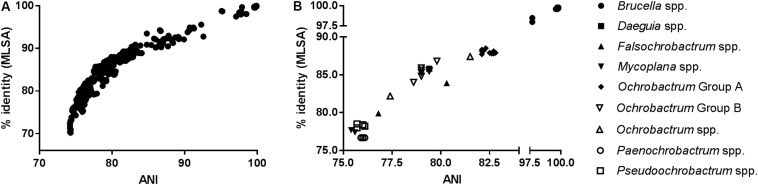
Association between sequence similarity indices from WGS (average nucleotide identity, ANI) and MLSA (percentage nucleotide identify of concatenated sequence) for *Brucellaceae* type strains. **(A)** The distribution of values for both indices, from all pairwise comparisons between members of the family (*n* = 903 comparisons). **(B)** Values for members of each genus, relative to the *Brucellaceae* type species (*B. melitensis* NCTC 10094^T^).

As anticipated, measures of sequence similarity for WGS (percentage ANI) and MLSA (percentage nucleotide identity) showed very high levels of agreement [Figure 1A; semi-logarithmic regression: *Y* = −283.2 + 193^∗^log(X); *R*^2^: 0.89]. The relationship between genomic ANI and MLSA nucleotide identity was further investigated by comparing their values for all species, relative to the *Brucellaceae* type species *B. melitensis* NCTC 10094^T^ (Figure 1B). Most notable was the very large dispersion of *Ochrobactrum* species, which were split into distinct groups by both metrics (Groups A and B as defined in section “Phylogenetic Analyses Based on WGS”), with one outlier (*O. endophyticum*) highly divergent from other members of the genus. Similarly, the two *Falsochrobactrum* species were characterized by very different levels of diversity relative to *B. melitensis*.

**FIGURE 2:**
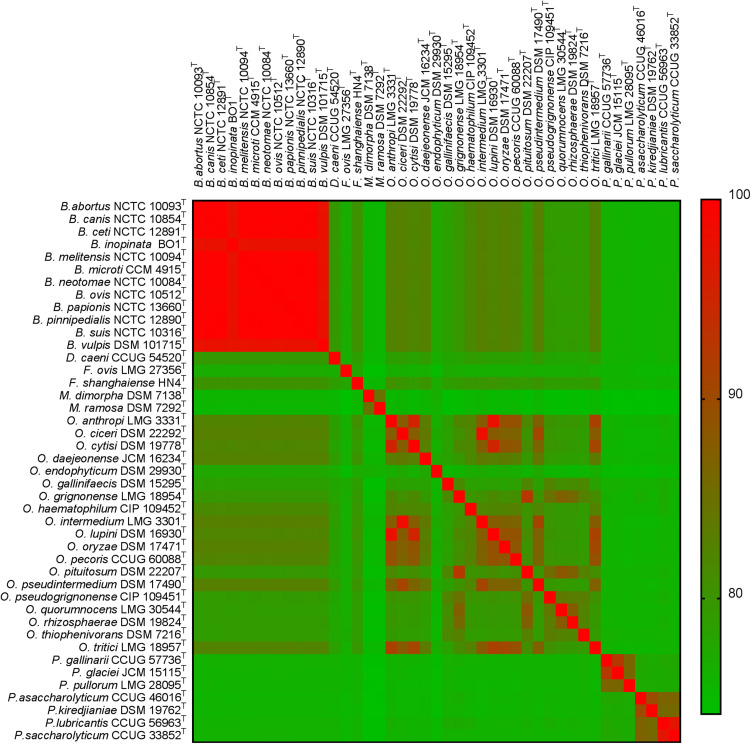
Heatmap of genomic average nucleotide identity (ANI) values for pairwise comparisons between all type strains of the family *Brucellaceae* (*n* = 43).

Further investigation of genomic ANI values (Figure 2) demonstrated that all pairwise comparisons between species within the genus *Brucella* exceeded 97.5%. Furthermore, a number of other intra-generic pairings with very high ANI values were identified. Specifically, within the genus *Ochrobactrum*, *O. ciceri* and *O. intermedium* were identified as sharing 98.5% genomic ANI, whilst *O. anthropi* and *O. lupini* (the latter already identified as a homotypic synonym of the former) shared 97.9% ANI. Outside of the *Brucella* and *Ochrobactrum* genera, the highest ANI value observed was between *Pseudochrobactrum lubricantis* and *P. sacchrolyticum* (97.1%).

### Phylogenetic Analysis of *Brucellaceae* Type Strains

#### Phylogenetic Analyses Based on WGS

**FIGURE 3:**
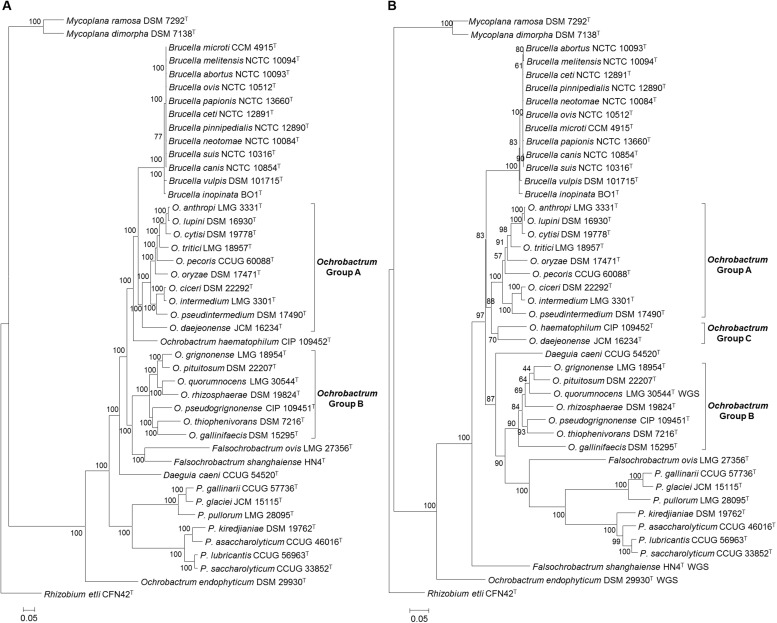
Phylogenetic relationship between type strains of the family *Brucellaceae* (*n* = 43), based on WGS **(A)** and MLSA **(B)**. In both cases the evolutionary history was inferred using the maximum likelihood method, based on the general time reversible model. Node labels give percentage bootstrap support. The scale bar shows the number of base substitutions per site. *Rhizobium etli* (CFN42^T^) was used to root the phylogenetic tree.

Maximum likelihood analysis based on the core genome alignment from *Brucellaceae* type strains resulted in an almost fully resolved phylogeny (Figure 3A), with the only nodes lacking full bootstrap support sitting within the genus *Brucella*. Nonetheless, the monophyly of this genus was highly supported. The genus *Ochrobactrum* was identified as polyphyletic. A sister group to the *Brucella* consisted of 10 *Ochrobactrum* species (Figure 3A: Group A), containing *O. anthropi* – *O. lupini* – *O. cytisi* – *O. tritici* – *O. pecoris* – *O. oryzae* – *O. ciceri* – *O. intermedium* – *O. pseudintermedium* – *O. daejeonense*. A single species (*O. haematophilum*), branched basally, to form a sister group to the clade containing these two taxa (*Ochrobactrum* Group A and *Brucella* spp.). A second group of seven *Ochrobactrum* species (Figure 3A: Group B) formed a sister group to the *Brucella* and all other *Ochrobactrum* species (with the exception of *O. endophyticum*). This clade was comprised of *O. grignonense* – *O. pituitosum* – *O. quorumnocens* – *O. rhizosphaerae* – *O. pseudogrignonense* – *O. thiophenivorans* – *O. gallinifaecis*.

Maximum likelihood phylogeny based on WGS data indicated that *Falsochrobactrum* spp. formed a sister clade to *Ochrobactrum* Group B. Further phylogenies were estimated using the WGS dataset (both with and without additional outgroups), removing each species in turn (*F. ovis* and *F. shanghaiense*). The phylogenies produced were identical in their placement of the two *Falsochrobactrum* species, which were individually placed as a sister taxon to *Ochrobactrum* Group B (Supplementary Figure S1). The single *Daeguia* species (*D. caeni*) branched basally to *Brucella* spp., *Ochrobactrum* Groups A and B and *Falsochrobactrum* spp. Both *Paenochrobactrum* spp. and *Pseudochrobactrum* spp. formed well-supported sister taxa in a single clade. A single *Ochrobactrum* species (*O. endophyticum*) branched basally to all members of the family, other than *Mycoplana* spp. (Figure 3A). Maximum likelihood phylogenetic analysis incorporating eight outgroup strains from the wider *Rhizobiales* indicated that *Mycoplana* spp. were placed with type strains of *Sinorhizobium meliloti* DSM 30135^T^ and *Ensifer* spp. (*E. sojae* DSM 26426^T^ and *E. adhaerens* ATCC 33212^T^), of the family *Rhizobiaceae*, rather than with members of the *Brucellaceae* (Supplementary Figure S1).

#### Phylogenetic Analysis Based on MLSA

Phylogenetic analyses based on concatenated sequences (3,004 bp) for all *Brucellaceae* type strains demonstrated that the pan-family MLSA scheme was able to distinguish all members of the taxon, with reasonable levels of bootstrap support in the majority of cases (Figure 3B). A single strongly supported clade containing all currently recognized *Brucella* species was evident. The species of *Ochrobactrum* included in the analysis of concatenated sequences formed three distinct clades. The first of these clades (Figure 3B: Group A) consisted of nine species, containing the grouping of *O. anthropi* – *O. lupini O. cytisi* – *O. tritici* – *O. oryzae* – *O. pecoris* – *O. ciceri* – *O. intermedium* – *O. pseudintermedium*. However, the node separating *O. pecoris* from other members of this clade was weakly supported. A second *Ochrobactrum* clade (Figure 3B: Group B) contained seven species (*O. grignonense* – *O. pituitosum* – *O. quorumnocens* – *O. rhizosphaerae* – *O. pseudogrignonense* – *O. thiophenivorans* – *O. gallinifaecis*) and grouped more closely with a clade containing *Pseudochrobactrum* spp., *Paenochrobactrum* spp. and *Falsochrobactrum ovis* than with the *Ochrobactrum* Group A clade. Bootstrap support for nodes within this grouping of seven *Ochrobactrum* species was generally low, with the exception of the nodes dividing *O. gallinifaecis* from all others, and dividing *O. thiophenivorans* from *O. pseudogrignonense*. A third clade of two *Ochrobactrum* species (Figure 3B: Group C: *O. daejeonense* and *O. haematophilum*) formed a distinct sister clade to Group A, though with only moderate bootstrap support. A well-supported clade contained *Pseudochrobactrum* spp., *Paenochrobactrum* spp. and *Falsochrobactrum ovis*, with *F. ovis* branching basally to form a sister taxon to the other two genera. The recently described *F. shanghaiense*, however, branched basally to all other *Brucellaceae* type strains, with the exception of a single *Ochrobactrum* species (*O. endophyticum*) and *Mycoplana* spp. *Daeguia caeni* was placed as a sister taxon to *Ochrobactrum* Group B and *Pseudochrobactrum* spp., *Paenochrobactrum* spp. and *F. ovis.*

#### Phylogenetic Analyses Based on Individual MLSA Loci and 16S rRNA Sequence

Maximum-likelihood phylogenies for each locus are shown in Supplementary Figure S2. In all cases, a single clade containing *Brucella* type species, including divergent atypical species (*B. inopinata* and *B. vulpis*) was identified, with a high level of support (bootstrap values of 100% for all loci). Only one locus (*trpE*) indicated the existence of a monophyletic *Ochrobactrum* clade. All other loci indicated that the genus was polyphyletic. A group of species, corresponding to *Ochrobactrum* Group A (as identified in analysis of both WGS and MLSA datasets), was evident for all loci except *acnA*. Within *Ochrobactrum* Group A, *O. anthropi* and *O. lupini* were consistently shown to be very closely related, with *O. cytisi* also consistently grouping with the former species, but somewhat divergent. Similarly, *O. ciceri* and *O. intermedium* were consistently identified as highly similar, with *O. pseudintermedium* forming a slightly divergent sister taxon. The relative position of the seven species within *Ochrobactrum* Group B (as identified in analysis of both WGS and MLSA datasets) was not consistent between loci, and generally had low levels of bootstrap support. Both *O. daejeonense* and *O. haematophilum* were inconsistently placed, and often weakly supported, in analyses of data from individual loci. Analysis of individual MLSA loci consistently identified well supported *Pseudochrobactrum* and *Paenochrobactrum* groups, as sister taxa. The position of the two *Falsochrobactrum* species was variable, however, and they were not placed in a unified taxon in any single locus analysis. The placement of the single *Daeguia* species was highly variable in analyses based on individual loci, and frequently had low levels of bootstrap support. In the majority of cases *O. endophyticum* branched basally to all members of the family except *Mycoplana* spp., and clearly distinct from other *Ochrobactrum* species. Finally, data from individual loci other than *gpd* identified the two *Mycoplana* species as branching basally to all other members of the family.

As observed in previous studies, analysis of almost full-length 16S rRNA sequences from *Brucellaceae* type strains did not provide sufficient resolution to correctly place species, even at the genus level (Supplementary Figure S2).

### Atypical *Brucella* Isolates

**FIGURE 4:**
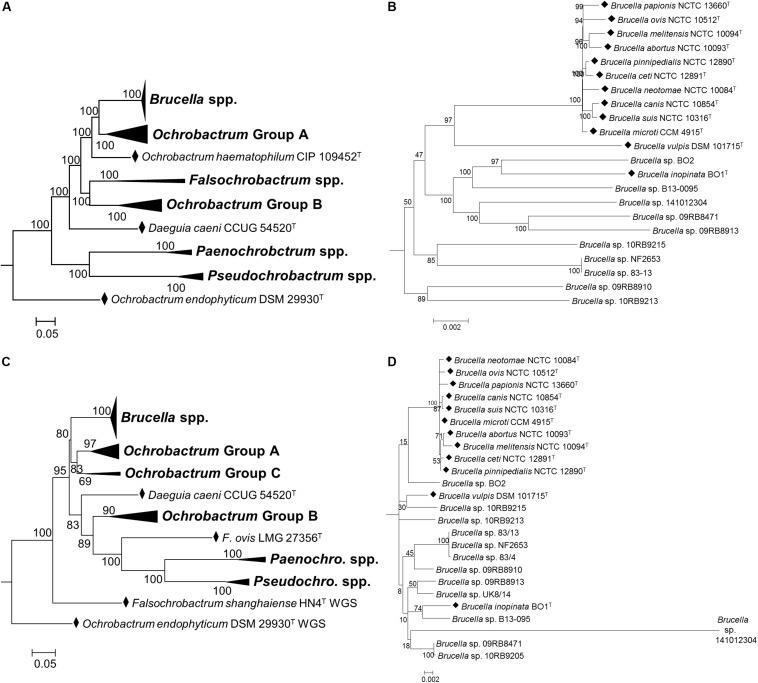
Phylogenetic relationship between type strains of the family *Brucellaceae* (and selected atypical *Brucella* sp. strains), based on WGS **(A,B)** and MLSA **(C,D)**. Panels **(A,C)** show the phylogeny inferred for the entire family, whilst panels **(B,D)** show only the *Brucella* spp. clade. Type strains are denoted by a black diamond (◆). In both cases the evolutionary history was inferred using the maximum likelihood method, based on the general time reversible model. Node labels give percentage bootstrap support. The scale bar shows the number of base substitutions per site. *Rhizobium etli* (CFN42^T^) was used to root the phylogenetic tree (not shown).

Inclusion of atypical *Brucella* isolates with type strains of the family *Brucellaceae* clearly identified them as belonging within the genus *Brucella*, with maximum bootstrap support for a monophyletic genus (Figures 4A,C). Additionally, both WGS and MLSA based approaches identified the six classical *Brucella* species, plus the more recently described *B. ceti*, *B. pinnipedialis*, *B. microti* and *B. papionis* as forming a well-supported group of ten core species, with limited diversity between them (Figures 4B,D). Outside of these core species, the basal branching order of atypical *Brucella* species and strains within the genus was relatively weakly supported by both WGS and MLSA datasets, with low bootstrap values for several nodes (Figures 4B,D). Amongst these isolates there were only a small number of groupings well supported by bootstrap values using MLSA data. Strains from Australian rodents (83/4, 83/13 and NF2653) formed a well-supported clade (Figure 4D), as did two strains from African bullfrogs (*Brucella* sp. 09RB8471 and 10RB9205). Based on MLSA data the human-associated strain *B. inopinata* formed a moderately well-supported group with a novel isolate from an amphibian (*Brucella* sp. B13-095), and distinct from the other atypical human isolate *Brucella* sp. BO2. In contrast, WGS based analysis identified these human associated isolates as sister taxa, with the amphibian isolate *Brucella* sp. B13-095 branching basally (Figure 4B). Branch lengths based on MLSA data indicated that *Brucella* sp. 141012304, isolated from a cartilaginous fish, was highly divergent from other atypical strains. Branch lengths of atypical *Brucella* strains calculated from WGS data, however, indicated a more consistent level of divergence from core species.

### Field Isolates

**FIGURE 5:**
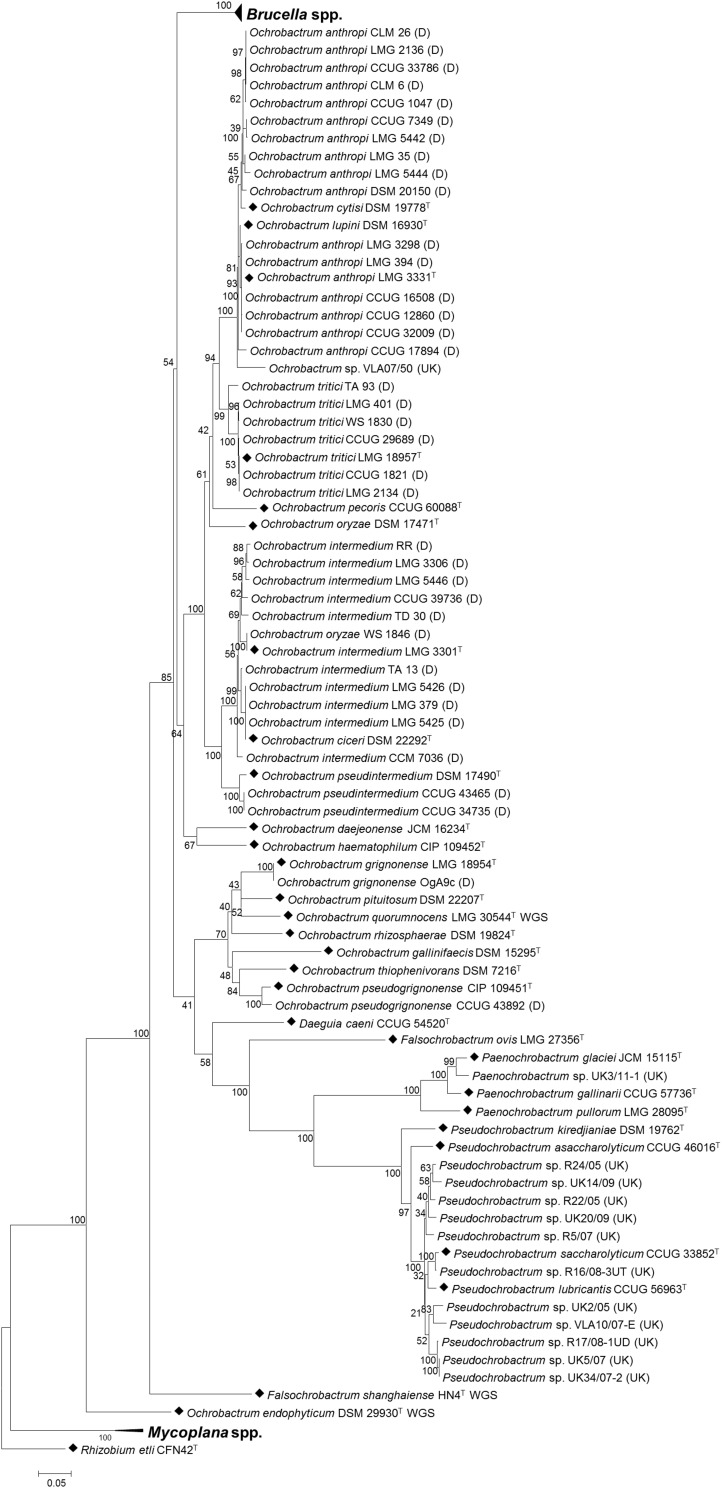
Phylogenetic relationship between type strains of the family *Brucellaceae* (*n* = 43) and field strains (*n* = 50), based on multi-locus sequence analysis. Type strains are denoted by a black diamond (◆), whilst strains from Bundeswehr Institute of Microbiology, Germany are denoted (D) and field strains from the Animal and Plant Health Agency, United Kingdom are denoted (UK). The evolutionary history was inferred using the maximum likelihood method, based on the general time reversible model. Node labels give percentage bootstrap support. The scale bar shows the number of base substitutions per site. *Rhizobium etli* (CFN42^T^) was used to root the phylogenetic tree.

In order to assess the value of the pan-family MLSA scheme in placing field isolates, subsequent analysis incorporated panels of field isolates, alongside type strains of all species within the *Brucellaceae*. This demonstrated that the MLSA scheme assigned field isolates to phylogenetic locations largely consistent with their identities assigned on the basis of phenotypic, *recA* and/or 16S rRNA sequence analysis (Figure 5).

Isolates previously identified as *Ochrobactrum anthropi* formed a well-supported clade containing the type strains of *O. anthropi* – *O. cytisi* and *O. lupini*. However, isolates did not fall into three separate clusters corresponding to the three species but rather represented a continuum of diversity. Only five *O. anthropi* field strains clustered with the type strain for the species. A further ten *O. anthropi* field strains grouped more closely with *O. cytisi* although with low levels of bootstrap support for the node separating them from the well supported *O. anthropi – O. lupini* cluster. A single field isolate (VLA07/50) grouped within the *O. anthropi* – *O. cytisi* and *O. lupini* clade with a high degree of bootstrap support, but appeared to be sufficiently divergent from other strains within the cluster to potentially represent a novel species. Interestingly, this isolate originated from a harbor porpoise (*Phocoena phocoena*) and hence may represent a marine or marine-mammal associated strain.

A sister clade to the *O. anthropi* – *O. cytisi* and *O. lupini* grouping contained the type strain of *O. tritici* and all field isolates identified as belonging to this species. A sister clade to the grouping of *O. anthropi* – *O. cytisi* – *O. lupini* and *O. tritici* contained the type species of *O. pseudintermedium* – *O. ciceri* and *O. intermedium* and associated field isolates. All field isolates of *O. pseudintermedium* grouped with their respective type strain (DSM 17490^T^). In the case of *O. intermedium*, five of the ten field isolates grouped most closely with the type strain (LMG 3301^T^), though with a continuum of diversity from the parent species. Four *O. intermedium* field strains clustered more closely with the *Ochrobactrum ciceri* type strain (DSM 22292^T^). A single *O. intermedium* field strain (CCM 7036) branched basally to the cluster containing type species of *O. intermedium* – *O. ciceri* and associated field isolates, indicating additional diversity within this group that may merit description of further species. A single field isolate originally identified as *O. oryzae* (WS 1846), clustered with the *O. intermedium* type strain and field isolates. The location of the type strain for this species (DSM 17471^T^) was relatively poorly supported.

A single field isolate grouped with the type strains of both *O. grignonense* and *O. pseudogrignonense* (field isolates OgA9c and CCUG 43892, respectively). In the case of the former (OgA9c), this strain was isolated simultaneously to the type strain, and from the same material (Lebuhn et al., 2000) which is reflected in their sequence homology. Strain CCUG 43892 was isolated independently from the type strain of *O. pseudogrignonense* (CIP 109451^T^), but formally described at the same time (Kämpfer et al., 2007a) due to their phenotypic and genetic similarity. This is reflected in the high level of bootstrap support for their placement together, though branch lengths indicate a degree of sequence divergence in the six MLSA loci described here.

With the exception of a single strain, United Kingdom field isolates (obtained via surveillance, for exclusion as *Brucella*), were identified as either *Pseudochrobactrum* sp. or *Paenochrobactrum* sp. by 16S sequencing (Table 3). *Pseudochrobactrum* sp. field strains formed a diverse cluster with the type strains of *P. lubricantis* and *P. saccharolyticum* suggesting these species poorly reflect actual diversity of these bacterial groups. In particular, a group of five field isolates within this grouping formed two distinct and well-supported clusters of two and three isolates, respectively. The single *Paenochrobactrum* sp. field strain (UK3/11-1) grouped in a single well-supported clade with type strains of the three described *Paenochrobactrum* species, and was identified as most closely resembling *P. glaciei*.

## Discussion

In this study, we have investigated phylogenetic relationships within the *Brucellaceae*, using a comprehensive WGS dataset incorporating all currently described species and a pan-family MLSA approach developed to be compatible with existing *Brucella* schemes.

Increasingly, WGS approaches are being used to generate data for phylogenetic placement of novel bacterial isolates. Nonetheless, the pan-family MLSA approach developed here remains valuable, in providing a defined list of genomic targets for future phylogenetic analysis and a comprehensive database of both reference and field isolates for comparison. Additionally, whilst the generation of WGS data for bacterial isolates is increasing, such data is absent for a large number of strains described before the approach became widespread. Again, the data generated in the current work will permit their integration into future multi-gene analyses incorporating results derived from both whole genome and “first-generation” sequencing methods. As an example of this approach, Krzyzanowska et al. (2019) recently described a putative novel *Ochrobactrum* species (*Ochrobactrum quorumnocens* sp. nov.) isolated from the potato rhizosphere. These authors applied a comparative genomic analysis with related *Ochrobactrum* type strains, using WGS data from the novel isolate and six existing species. These data were used to apply an MLSA based approach using concatenated nucleotide sequences of three genes: 16S rRNA gene, *groEL* and *gyrB*. Retrieval of data for the genes adopted in the current work would permit the comparison of this novel strain to a wider diversity of species within the *Brucellaceae*.

Our work provides an additional tool to understand strains where conventional diagnostic approaches are confounded. Recently described “atypical” *Brucella* sp. isolates can be confused with *Ochrobactrum* sp. by diagnostic approaches such as API 20NE tests (Scholz et al., 2010; Tiller et al., 2010b; Soler-Lloréns et al., 2016). These isolates can also confound identification by matrix-assisted laser desorption/ionization time-of-flight (MALDI-TOF) mass spectrometry (MS), an increasingly widely used front-line tool in bacterial diagnostics (Cunningham and Patel, 2013). Thus there are examples where atypical *Brucella* either return no reliable identification or are misidentified as *B. melitensis* (Whatmore et al., 2015; Mühldorfer et al., 2017). The pan-family MLSA approach applied here clearly identified that all atypical strains incorporated into the analysis are much more closely related to *Brucella* than to *Ochrobactrum*.

The WGS and MLSA approaches applied here are of value in informing the ongoing debate concerning *Brucella* taxonomy and evolution, and allow accurate placement and understanding of the relationships of these organisms with extant isolates. Both methods identified a monophyletic *Brucella* clade, with a highly conserved group of ten core species and greater diversity in recently identified atypical strains. Basal branches in this clade were, however, relatively weakly supported by both methods. Nonetheless, based on currently accepted practices for defining species based on genomic ANI values (Varghese et al., 2015) all *Brucella* strains incorporated into our analyses would represent a single species. *Brucella* taxonomy continues to be controversial both at the intra-genus level and in terms of relationships with closely related species. The previous re-designation of the then six original “core” *Brucella* species as a single species based on genetic homogeneity (Verger et al., 1985) was reversed, based on community preference for a nomenclature that reflected clear differences in epidemiology and pathogenic potential of the nomenspecies (Osterman and Moriyón, 2006). The current study emphasizes the genetic homogeneity of the expanded core group, in contrast to the greater diversity seen in the recently emerged atypical *Brucella*. Attempts to describe emerging amphibian isolates as new species failed because of the heterogeneity of the isolates (Al Dahouk et al., 2017) and their placement here confirms that there are currently few distinct clusters of isolates among the atypical *Brucella* that would readily facilitate description of any well-defined new species. A more pragmatic, though not cladistic, solution may be to update the species description for *B. inopinata*, originally based on only a single isolate (BO1), taking into account the large number of recently described strains, and potentially classify all atypical *Brucella* as *B. inopinata*. Arguments that these isolates should not be members of the genus *Brucella*, as currently defined, are not supported by the genetic relationships observed here, as they clearly remain well separated from the closest *Ochrobactrum* isolates.

The current work supports previous findings, based on single-gene, multi-locus and whole genome analyses, which indicated that the genus *Ochrobactrum* represents a polyphyletic grouping (Kämpfer et al., 2013; Aujoulat et al., 2014; Leclercq et al., 2019). Our study provides the most comprehensive analysis to date of the composition of these groupings, and their relationships to other members of the family. Both WGS and MLSA based analyses identified two main clusters, with *Ochrobactrum* Group A (*O. anthropi*; *O. lupini, O. cytisi*; *O. tritici*; *O. pecoris*; *O. oryzae*; *O. ciceri*, *O. intermedium, O. pseudintermedium*) forming a well-supported sister taxa to all *Brucella* species. The position of two further species (*O. daejeonense* and *O. haematophilum*) diverged slightly between the two methods, though both were placed with the *Brucella*-*Ochrobactrum* Group A clade (with WGS indicating *O. haematophilum* to branch basally to this group). A further *Ochrobactrum* clade (Group B) contained seven species (*O. gallinifaecis* – *O. quorumnocens* – *O. rhizosphaerae* – *O. grignonense* – *O. pituitosum* – *O. pseudogrignonense* – *O. thiophenivorans*) and formed a sister taxon to the *Brucella* – *Ochrobactrum* Group A clade in WGS based analyses (An *Ochrobactrum* group of the same composition was identified in MLSA based analysis, but in this case it was placed as a sister taxon to the clade containing *Pseudochrobactrum* spp., *Paenochrobactrum* spp. and *Falsochrobactrum ovis*). Recently, Leclercq et al. (2019) applied a phylogenomic approach to identify similar polyphyletic *Ochrobactrum* clades to those identified here. These authors suggest that there is a separation in the ecological niche of species belonging to *Ochrobactrum* groups A and B, with the former characterized by human and animal derived isolates, and the latter mainly composed of environmental and plant-associated isolates. Grouping type strains by their source of isolation did not reveal any association between *Ochrobactrum* groups and human/animal origin of isolation in the current study (Fisher’s exact test, *P* > 0.05 for both WGS and MLSA data).

Two *Ochrobactrum* species (*O. daejeonense and O. haematophilum*) were inconsistently placed, and often weakly supported, in analyses of data from individual loci. Examples of incongruence in placement between single gene trees of *Ochrobactrum* species are supportive of previous suggestions that horizontal gene transfer may be a driving force in the evolution of both atypical *Brucella* (Scholz et al., 2016b) and other free-living members of the *Brucellaeae* (Aujoulat et al., 2014). This is in contrast to the situation in the core *Brucella* which have been considered largely clonal with only rare horizontal gene transfer events contributing to speciation (Whatmore et al., 2007; Wattam et al., 2014).

Analyses in the current work (WGS, MLSA, individual gene and 16S rRNA based) indicated that *O. endophyticum* (DSM 29930^T^) is not located within the *Ochrobactrum* groupings described above, but instead forms a distinct taxon, basal to other members of the family (with the exception of *Mycoplana* spp., though see below). Analysis of WGS data incorporating additional outgroup species (Supplementary Figure S1) also indicated that *O. endophyticum* remained basal to other members of the *Brucellaceae*, but was more closely related to them than the nearest taxonomic neighbors included in the analysis (*Mesorhizobium* spp. and *Aquamicrobium aerolatum*). Clarification of the true taxonomic position of *O. endophyticum* will require the incorporation of additional strains outside of the *Brucellaceae*, but it appears that it may require amendment.

Analysis of WGS and MLSA data from *Brucellaceae* type strains identified *O. anthropi* and *O. lupini* to be closely related, consistent with a recent analysis based on WGS data, which proposed that *O. lupini* should be re-classified as a later heterotypic synonym of *O. anthropi* (Gazolla Volpiano et al., 2019). These authors also highlighted the high level of similarity observed between *O. anthropi*/*O. lupini* and *O. cytisi*, and recommended WGS of the type strain for the latter species, to clarify its position. Incorporation of WGS data for *O. cytisi* DSM 19778^T^ in the current study indicated that it is correctly identified as an independent species (adopting the threshold of 96.5% proposed by Varghese et al., 2015) with genomic ANI values of 95.2% and 95.1% relative to *O. anthropi*/*O. lupini*, respectively. Leclercq et al. (2019) drew the same conclusion based on sequencing of a non-type strain of *O. cytisi* (IPA7.2). High levels of similarity, consistent with possible re-classification as a single species were observed between type strains of *O. ciceri* and *O. intermedium* (genomic ANI of 98.5%) and *P. lubricantis* and *P. saccharolyticum* (genomic ANI of 97.1%).

Furthermore, it is also clear from the inclusion of field isolates into multi-locus analysis of *Brucellaceae* that there is much previously unrecognized diversity that is not reflected in currently identified species. Notable diversity was evident within the *O. anthropi* – *O. cytisi* – *O. lupini*, *O. intermedium* – *O. ciceri* and *P. saccharolyticum* – *P. lubricantis* groupings, resulting in a lack of clear monophyletic lineages for any of these taxa once field isolates were incorporated.

Basal branching orders within type strain phylogenies were largely consistent between WGS and MLSA based approaches (Figure 3). One instance where this was not the case, however, was in the placement of *Falsochrobactrum*. Both *F. ovis* and *F. shanghaiense* were placed as a sister taxon to *Ochrobactrum* Group B in analyses based on WGS data. Conversely, in analyses using MLSA, the taxon was split, with *F. ovis* placed as a sister taxon to the *Paenochrobactrum* spp., and *Pseudochrobactrum* spp. group, and *F. shanghaiense* placed basal to all member of the family other than *O. endophyticum* and *Mycoplana* spp. Leclercq et al. (2019) reported inconsistent placement of *Falsochrobactrum* spp. using a whole genome dataset, with the location differing when both species were included relative to when either was included independently. However, neither placement was fully supported by bootstrap values in their analyses. The inclusion of a more comprehensive panel of related species and, potentially, the larger number of loci employed, provides greater confidence in the placement of these species in WGS analyses from the present study, as evidenced by the level of bootstrap support achieved. Nonetheless, it is clear from levels of sequence similarity observed within this genus (both genomic ANI and MLSA nucleotide identity) that these two species are highly divergent (Figure 1 and Table 4).

Finally, incorporation of additional outgroup strains in analyses of WGS data in the current work has demonstrated that *Mycoplana* spp. were placed with maximum bootstrap support within the family *Rhizobiaceae* rather than within the *Brucellaceae*. This is consistent with the findings of Leclercq et al. (2019) who incorporated a dataset encompassing the wider order *Rhizobiales* and demonstrated the same placement of *Mycoplana* spp. with *Ensifer* and *Sinorhizobium* species.

## Conclusion

The work presented here provides a comprehensive multi-gene analysis of the phylogeny of an expanding bacterial family, which includes a number of significant human and veterinary pathogens. It further demonstrates the importance of a multi-gene approach, be it WGS or MLSA based, in establishing robust relationships within this group. This work has demonstrated that the genus *Brucella* as currently described forms a well-separated monophyletic group, despite its on-going expansion to incorporate genetically diverse strains from a wide range of host species. Furthermore, the work confirms *Ochrobactrum* to be polyphyletic. The analysis of non-type strains of some members of *Brucellaceae* reveals that there remains significant genetic diversity not captured within existing species. The tools and databases developed in this study provide a valuable resource against which to place novel isolates and begin to identify new groups.

## Data Availability Statement

WGS data generated for this study can be found in the NCBI SRA database under BioProject PRJNA638390. MLSA data can be found in either the Brucella PubMLST database (https://pubmlst.org/brucella/) or in the NCBI GenBank database (accession numbers given in Supplementary Table S4).

## Author Contributions

RA analyzed the data and drafted the manuscript. JM and MK undertook experimental work and data analysis. HS provided strains and contributed to the manuscript. AW conceived of and designed the study and contributed to the manuscript. All authors contributed to the article and approved the submitted version.

## Conflict of Interest

The authors declare that the research was conducted in the absence of any commercial or financial relationships that could be construed as a potential conflict of interest.

## Footnotes

1 https://github.com/tseemann/nullarbor
