# Supplementary material for: Application of Whole Genome Sequencing and Pan-Family Multi-Locus Sequence Analysis to Characterize Relationships Within the Family Brucellaceae
Source: Front Microbiol. 2020 Jul 14;11:1329. doi: 10.3389/fmicb.2020.01329 (PMC7372191; doi:10.3389/fmicb.2020.01329)
Supplement: Supplementary file 1 [file Image_1.pdf]

Supplementary Figure S1

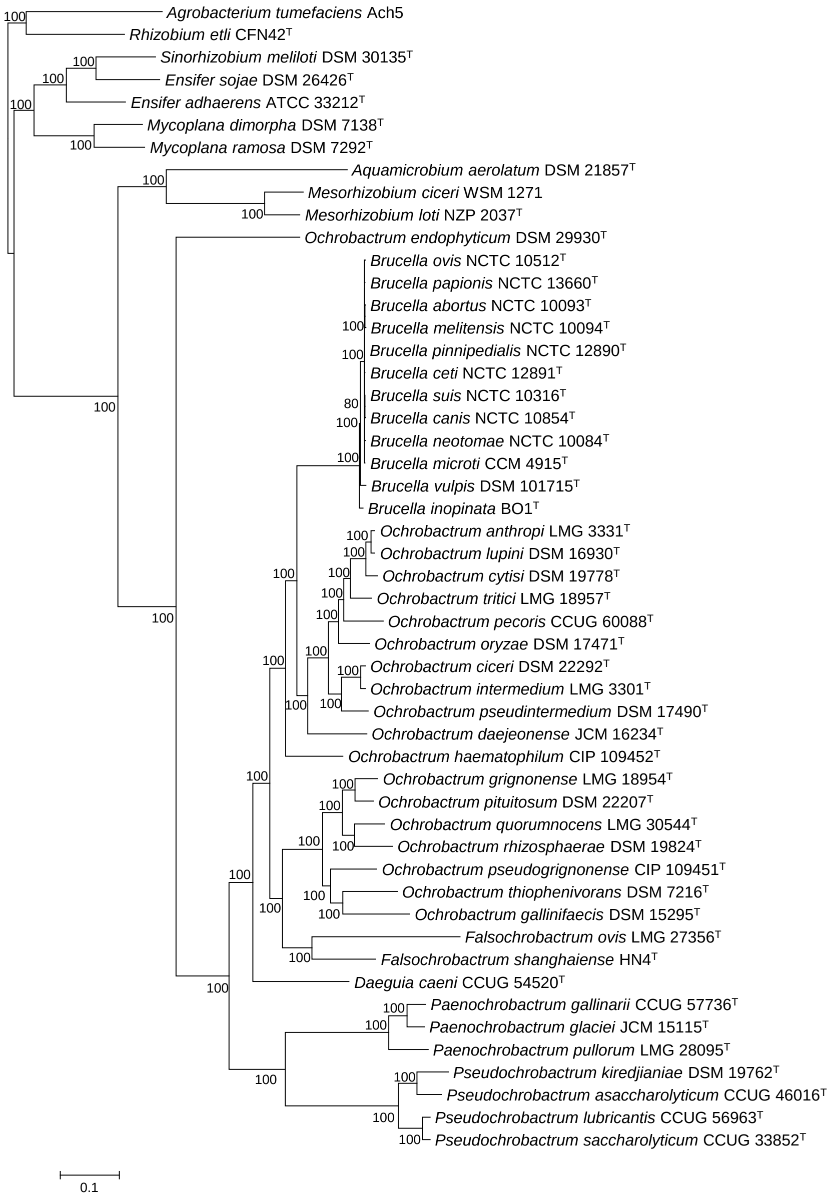

Figure S1.1 Phylogenetic relationship between type strains of the family *Brucellaceae* (n=43), incorporating outgroup strains from the wider *Rhizobiales*. The evolutionary history was inferred from a core genome alignment of 410 genes, using the maximum likelihood method, based on the general time reversible model. Node labels give percentage bootstrap support. The scale bar shows the number of base substitutions per site. The clade containing *Rhizobium etli* (CFN42^T) and *Agrobacterium tumefaciens* (Ach5) was used to root the phylogenetic tree.

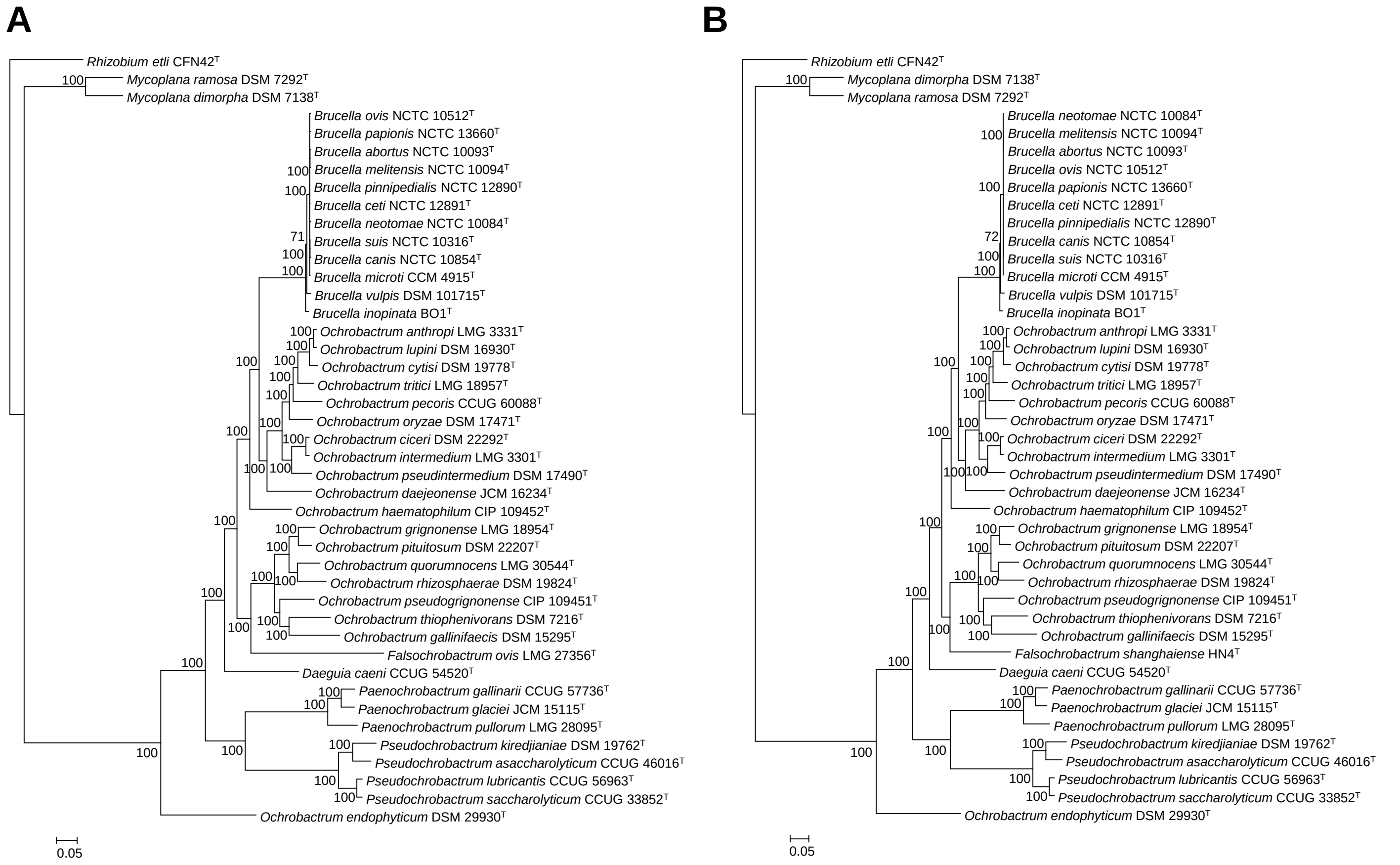

Figure S1.2 Phylogenetic relationship between type strains of the family *Brucellaceae* (n=43), sequentially excluding *F. shanghaiense* (A) and *F. ovis* (B). The evolutionary history was inferred from a core genome alignment of 450 genes using the maximum likelihood method, based on the general time reversible model. Node labels give percentage bootstrap support. The scale bar shows the number of base substitutions per site. *Rhizobium etli* (CFN42^{T}) was used to root the phylogenetic tree.

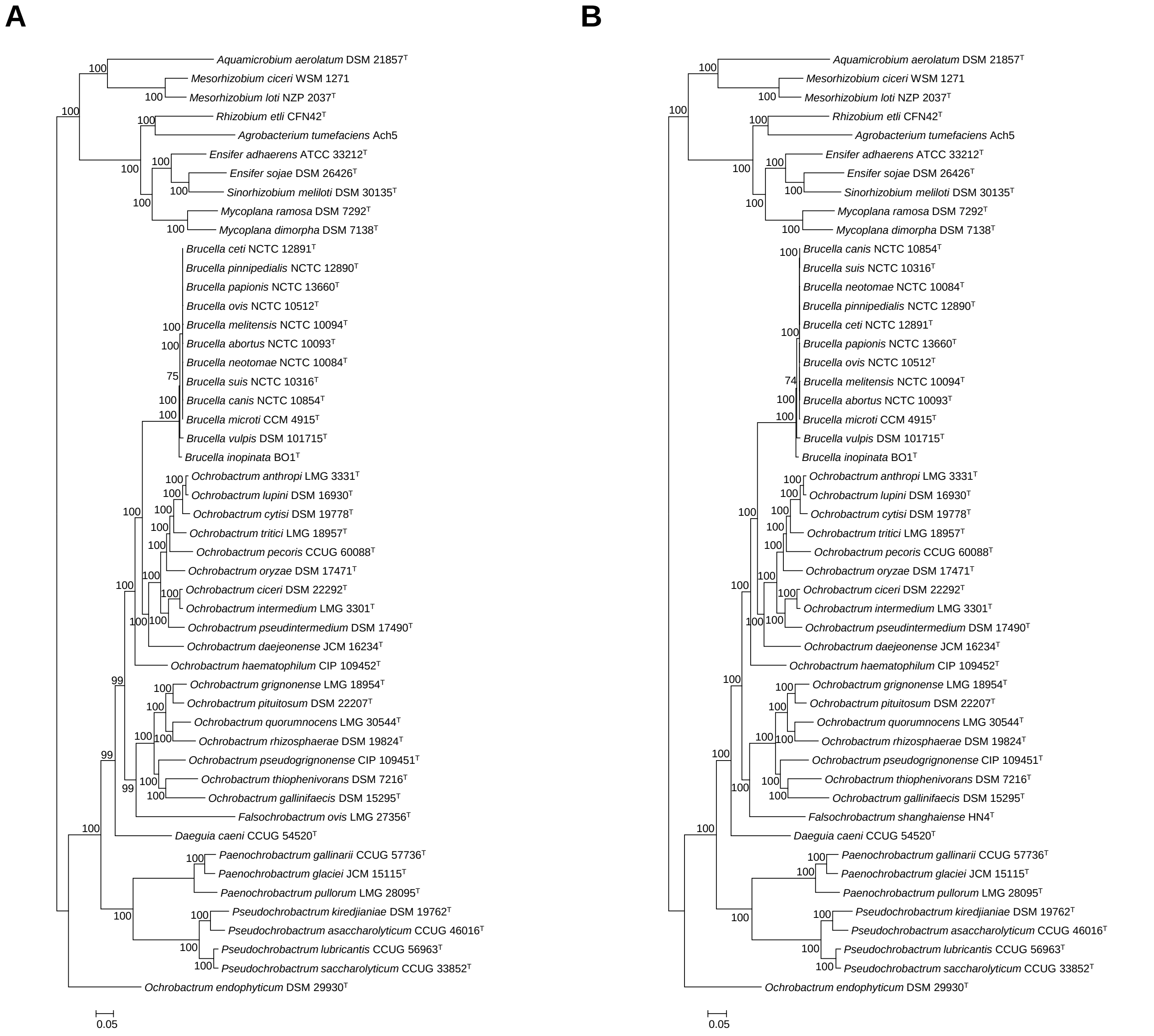

Figure S1.3 Phylogenetic relationship between type strains of the family *Brucellaceae* (n=43), incorporating outgroup strains from the wider *Rhizobiales*, sequentially excluding *F. shanghaiense* (A) and *F. ovis* (B). The evolutionary history was inferred from a core genome alignment of 410 genes using the maximum likelihood method, based on the general time reversible model. Node labels give percentage bootstrap support. The scale bar shows the number of base substitutions per site. The clade containing *Rhizobium etli* (CFN42^T) and *Agrobacterium tumefaciens* (Ach5) was used to root the phylogenetic tree.
