# Supplementary material for: Application of Whole Genome Sequencing and Pan-Family Multi-Locus Sequence Analysis to Characterize Relationships Within the Family Brucellaceae
Source: Front Microbiol. 2020 Jul 14;11:1329. doi: 10.3389/fmicb.2020.01329 (PMC7372191; doi:10.3389/fmicb.2020.01329)
Supplement: Supplementary file 2 [file Image_2.pdf]

Supplementary Figure S2

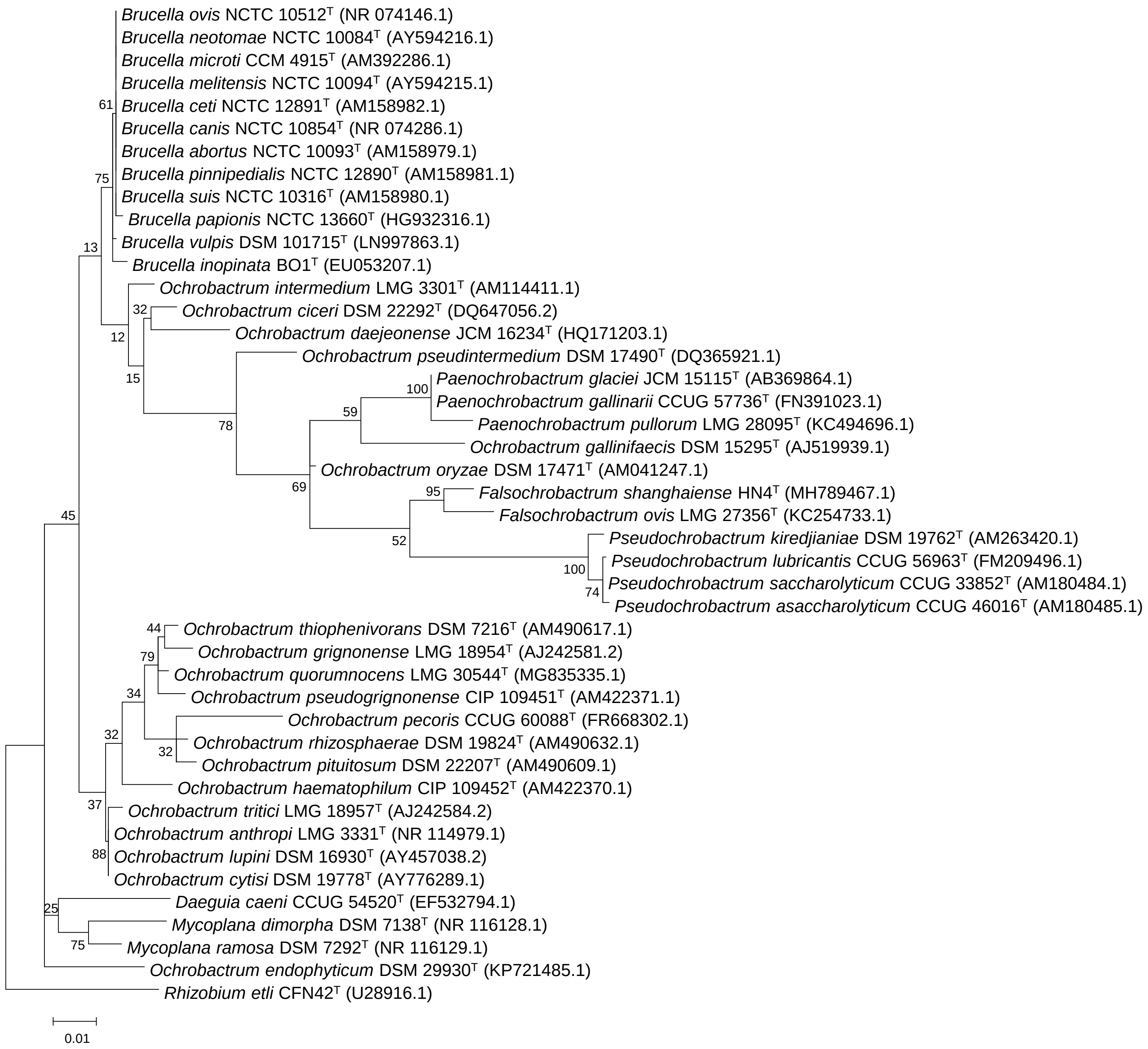

Figure S2.1 Phylogenetic relationship between type strains of the family *Brucellaceae* (n=43), based on analysis of 16 rRNA sequence. The evolutionary history was inferred using the maximum likelihood method, based on the general time reversible model. Node labels give percentage bootstrap support. The scale bar shows the number of base substitutions per site. *Rhizobium etli* (CFN42^T) was used to root the phylogenetic trees.

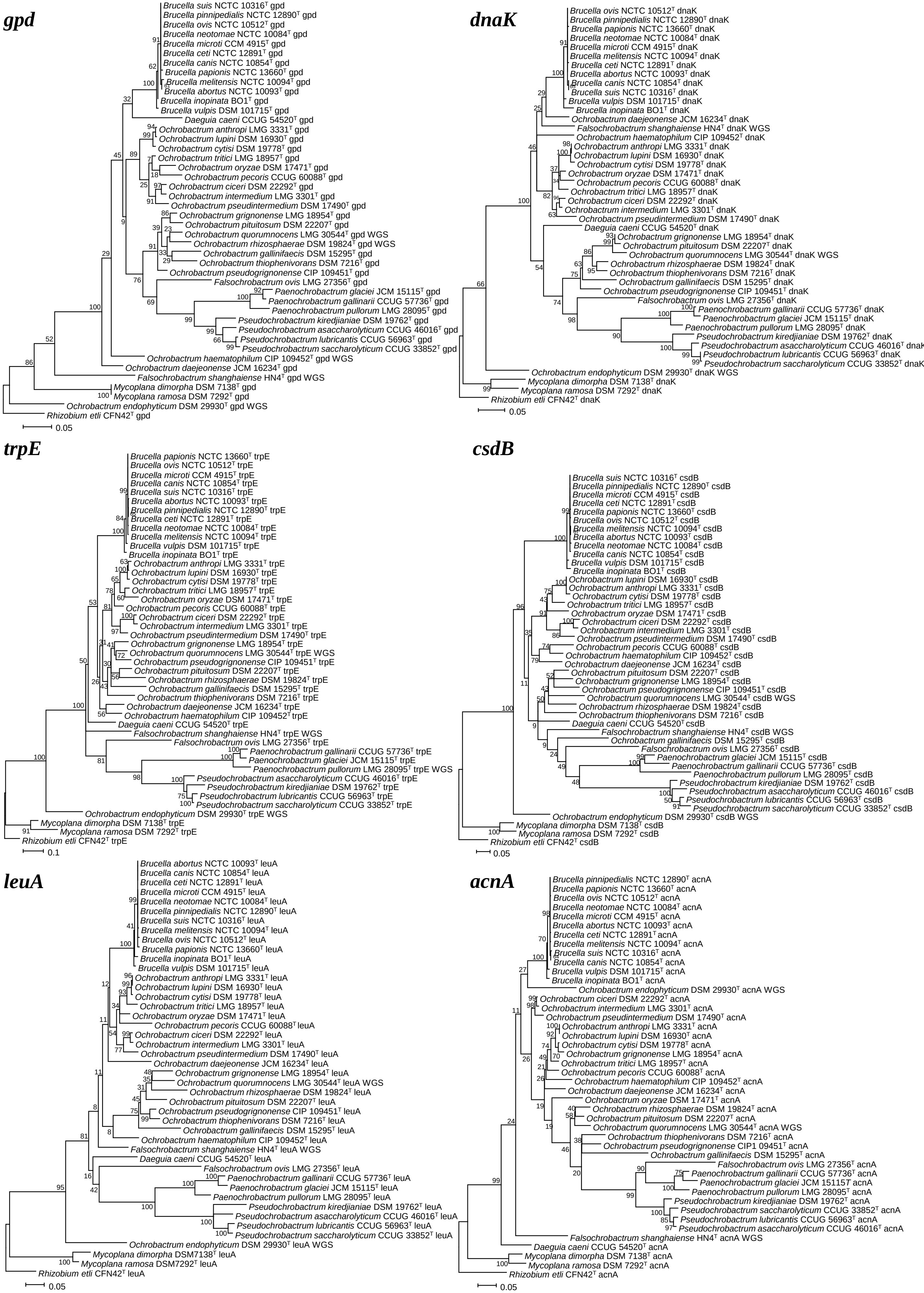

Figure S2.2 Phylogenetic relationship between type strains of the family *Brucellaceae* (n=43), based on analysis of individual MLSA loci. The evolutionary history was inferred using the maximum likelihood method, based on the GTR model. Node labels give percentage bootstrap support. The scale bar shows the number of base substitutions per site. *Rhizobium etli* (CFN42^{T}) was used to root the phylogenetic trees. Sequences retrieved from WGS data are annotated (WGS).
