# Supplementary material for: Application of Whole Genome Sequencing and Pan-Family Multi-Locus Sequence Analysis to Characterize Relationships Within the Family Brucellaceae
Source: Front Microbiol. 2020 Jul 14;11:1329. doi: 10.3389/fmicb.2020.01329 (PMC7372191; doi:10.3389/fmicb.2020.01329)
Supplement: Supplementary file 3 [file Table_1.pdf]

Supplementary Table S1

Table S1. *De novo* assembly statistics for *Brucellaceae* type strains for which whole genome sequencing data was generated within this study.

Species	Strain	% GC content	Length (bp)	Contigs	N50	CDS	rRNA	tRNA	Fold coverage
Daeguia caeni	CCUG 54520^T	56.2	3245719	127	85870	3030	3	48	91
Falsochrobactrum ovis	LMG 27356 ^{T}	50.4	3267899	43	295525	3122	3	45	36
Mycoplana dimorpha	DSM 7138^T	63.5	4590109	39	347281	4217	3	50	55
Mycoplana ramosa	DSM 7292^T	63.5	4466277	53	271800	4133	3	50	126
Ochrobactrum ciceri	DSM 22292^T	57.6	4571133	42	475114	4352	3	52	76
Ochrobactrum cytisi	DSM 19778^T	55.6	5792978	81	223698	5542	3	51	114
Ochrobactrum daejeonense	JCM 16234^T	58.3	4648313	86	149521	4447	3	52	130
Ochrobactrum endophyticum	DSM 29930^T	58.4	4932768	136	120234	4705	3	53	181
Ochrobactrum grignonense	LMG 18954^T	53.5	4948799	64	286645	4656	6	50	64
Ochrobactrum haematophilum	CIP 109452^T	56	5508453	69	216671	5213	3	53	57
Ochrobactrum lupini	DSM 16930^T	55.4	5591304	71	152284	5377	3	49	75
Ochrobactrum oryzae	DSM 17471^T	55.9	4773851	84	237924	4529	3	52	103
Ochrobactrum pituitosum	DSM 22207^T	53.1	5160499	47	377454	4949	3	51	88
Ochrobactrum pseudogrignonense	CIP 109451^T	53.4	5542201	79	281360	5313	3	51	73

Ochrobactrum rhizosphaerae	DSM 19824^T	52.8	4904656	47	347797	4628	3	47	114
Ochrobactrum thiophenivorans	DSM 7216^T	51.1	4369569	86	168741	4113	3	48	118
Paenochrobactrum gallinarii	CCUG 57736^T	49.2	3401076	113	150153	3150	3	44	56
Paenochrobactrum glaciei	JCM 15115^T	48.9	3703195	43	389832	3441	3	48	77
Paenochrobactrum pullorum	LMG 28095^T	48.6	3663848	57	201991	3454	3	53	29
Pseudochrobactrum asaccharolyticum	CCUG 46016^T	50.2	4648602	48	451575	4246	3	53	114
Pseudochrobactrum kiredjianiae	DSM 19762^T	50.4	4413605	63	461670	3934	3	49	136
Pseudochrobactrum lubricantis	CCUG 56963^T	51.9	4238784	60	439369	3930	3	48	111
Pseudochrobactrum saccharolyticum	CCUG 33852^T	51.2	3757080	25	730432	3361	3	47	76
