# Supplementary material for: Application of Whole Genome Sequencing and Pan-Family Multi-Locus Sequence Analysis to Characterize Relationships Within the Family Brucellaceae
Source: Front Microbiol. 2020 Jul 14;11:1329. doi: 10.3389/fmicb.2020.01329 (PMC7372191; doi:10.3389/fmicb.2020.01329)
Supplement: Supplementary file 4 [file Table_2.pdf]

Supplementary Table S2

Table S2.1 Primers used for amplification and sequencing of six loci included in the pan-family MLSA scheme

Locus name	Putative function	Primer direction	Primer sequence (5'-3')
gpd	glyceraldehyde 3-phosphate dehydrogenase	F	TGGAATGCACCGGCATYTTCA
		R	TCGSACATRCGGYTSGAGAAGC
dnaK	chaperone protein	F	TCGARGTGAAGTCSACCAAYG
		R	ACTTCTTSGTCGGGATCGTSG
trpE	anthranilate synthase	F	GGIGCICCRAARCTSTGGGC
		R	CGRATGCGIGAIGGYTTSCC
csdB	cysteine desulferase	F	GGMGGATSRTTRTAGGTGAC
		R	GARTAYGCCAAYGTSCATCG
leuA	2-isopropylmalate synthase	F	CGCTGTTCGGCAATGGCGAGC
		R	GCTGCAGCGAGTGYTCGGAAT
acnA	aconitate hydratase	F	CTGYGGYTTCTTCCCGGT
		R	GTCCCAKGCRTAGGTCTG

Table S2.2 Characteristics of the six loci included in the pan-family MLSA scheme, across the 43 *Brucellaceae* type species analysed

Locus	***Length (bp)***	***Mean % GC content***	***Polymorphic sites (%)***[a]	***π***
gpd	589	57.8	290 (49.24)	0.1403
dnaK	470	57.9	199 (42.34)	0.1256
trpE	486	57.6	260 (53.50)	0.1975
csdB	487	54.5	250 (51.33)	0.1780
leuA	482	54.5	237 (49.17)	0.1602
acnA	490	59.4	219 (44.69)	0.1412
Concatenated	3004	57.0	1453 (48.37)	0.1565

(a) Polymorphic sites observed amongst 43 *Brucellaceae* type strains analysed; π = nucleotide diversity
