# Supplementary material for: Application of Whole Genome Sequencing and Pan-Family Multi-Locus Sequence Analysis to Characterize Relationships Within the Family Brucellaceae
Source: Front Microbiol. 2020 Jul 14;11:1329. doi: 10.3389/fmicb.2020.01329 (PMC7372191; doi:10.3389/fmicb.2020.01329)
Supplement: Supplementary file 6 [file Table_4.pdf]

Supplementary Table S4

Table S4.1. NCBI GenBank accession numbers for MLSA data generated for type strains

Species	Strain ID	*gpd*	*dnaK*	*trpE*	*csdB*	*leuA*	*acnA*
B. abortus	NCTC 10093[T]	-[a]	-[a]	-[a]	-[a]	-[a]	-[a]
B. canis	NCTC 10854[T]	-[a]	-[a]	-[a]	-[a]	-[a]	-[a]
B. ceti	NCTC 12891[T]	-[a]	-[a]	-[a]	-[a]	-[a]	-[a]
B. inopinata	BO1[T]	-[a]	-[a]	-[a]	-[a]	-[a]	-[a]
B. melitensis	NCTC 10094[T]	-[a]	-[a]	-[a]	-[a]	-[a]	-[a]
B. microti	CCM 4915[T]	-[a]	-[a]	-[a]	-[a]	-[a]	-[a]
B. neotomae	NCTC 10084[T]	-[a]	-[a]	-[a]	-[a]	-[a]	-[a]
B. ovis	NCTC 10512[T]	-[a]	-[a]	-[a]	-[a]	-[a]	-[a]
B. papionis	NCTC 13660[T]	-[a]	-[a]	-[a]	-[a]	-[a]	-[a]
B. pinnipedialis	NCTC 12890[T]	-[a]	-[a]	-[a]	-[a]	-[a]	-[a]
B. suis	NCTC 10316[T]	-[a]	-[a]	-[a]	-[a]	-[a]	-[a]
B. vulpis	DSM 101715[T]	-[a]	-[a]	-[a]	-[a]	-[a]	-[a]
D. caeni	CCUG 54520[T]	MT637241	MT637267	MT637295	MT637322	MT637350	MT637378
F. ovis	LMG 27356 [T]	MT637242	MT637268	MT637296	MT637323	MT637351	MT637379
M. dimorpha	DSM 7138[T]	MT637243	MT637269	MT637297	MT637324	MT637352	MT637380
M. ramosa	DSM 7292[T]	MT637244	MT637270	MT637298	MT637325	MT637353	MT637381
O. anthropi	LMG 3331[T]	MT637245	MT637271	MT637299	MT637326	MT637354	MT637382
O. ciceri	DSM 22292[T]	MT637246	MT637272	MT637300	MT637327	MT637355	MT637383
O. cytisi	DSM 19778[T]	MT637247	MT637273	MT637301	MT637328	MT637356	MT637384
O. daejeonense	JCM 16234[T]	MT637248	MT637274	MT637302	MT637329	MT637357	MT637385
O. gallinifaecis	DSM 15295[T]	MT637249	MT637275	MT637303	MT637330	MT637358	MT637386
O. grignonense	LMG 18954[T]	MT637250	MT637276	MT637304	MT637331	MT637359	MT637387
O. haematophilum	CIP 109452[T]	-[b]	MT637277	MT637305	MT637332	MT637360	MT637388

O. intermedium	LMG 3301[T]	MT637251	MT637278	MT637306	MT637333	MT637361	MT637389
O. lupini	DSM 16930[T]	MT637252	MT637279	MT637307	MT637334	MT637362	MT637390
O. oryzae	DSM 17471[T]	MT637253	MT637280	MT637308	MT637335	MT637363	MT637391
O. pecoris	CCUG 60088[T]	MT637254	MT637281	MT637309	MT637336	MT637364	MT637392
O. pituitosum	DSM 22207[T]	MT637255	MT637282	MT637310	MT637337	MT637365	MT637393
O. pseudintermedium	DSM 17490[T]	MT637256	MT637283	MT637311	MT637338	MT637366	MT637394
O. pseudogrignonense	CIP 109451[T]	MT637257	MT637284	MT637312	MT637339	MT637367	MT637395
O. rhizosphaerae	DSM 19824[T]	- [b]	MT637285	MT637313	MT637340	MT637368	MT637396
O. thiophenivorans	DSM 7216[T]	MT637258	MT637286	MT637314	MT637341	MT637369	MT637397
O. tritici	LMG 18957[T]	MT637259	MT637287	MT637315	MT637342	MT637370	MT637398
P. gallinarii	CCUG 57736[T]	MT637260	MT637288	MT637316	MT637343	MT637371	MT637399
P. glaciei	JCM 15115[T]	MT637261	MT637289	MT637317	MT637344	MT637372	MT637400
P. pullorum	LMG 28095[T]	MT637262	MT637290	- [b]	MT637345	MT637373	MT637401
P. asaccharolyticum	CCUG 46016[T]	MT637263	MT637291	MT637318	MT637346	MT637374	MT637402
P. kiredjianiae	DSM 19762[T]	MT637264	MT637292	MT637319	MT637347	MT637375	MT637403
P. lubricantis	CCUG 56963[T]	MT637265	MT637293	MT637320	MT637348	MT637376	MT637404
P. saccharolyticum	CCUG 33852[T]	MT637266	MT637294	MT637321	MT637349	MT637377	MT637405

[a] MLSA data retrieved from Brucella PubMLST database

[b] Sequence supported by whole genome sequencing data

Table S4.2. NCBI GenBank accession numbers for MLSA data generated for atypical *Brucella* sp. strains

Strain ID	Source	Reference	*gpd*	*dnaK*	*trpE*	*csdB*	*leuA*	*acnA*
BO2	Human lung biopsy	Tiller et al., 2010a	- [a]	- [a]	- [a]	- [a]	- [a]	- [a]
83/13	Australian rodent	-	- [a]	- [a]	- [a]	- [a]	- [a]	- [a]
83/4	Australian rodent	-	- [a]	- [a]	- [a]	- [a]	- [a]	- [a]
NF2653	Australian rodent	Wattam et al., 2012	- [b]	- [b]	- [b]	- [b]	- [b]	- [b]
09RB8471	African bull frog (*Pyxicephalus edulis*)	Al Dahouk et al., 2017	- [a]	- [a]	- [a]	MT637406	MT637413	MT637420
09RB8910	African bull frog (*Pyxicephalus edulis*)	Al Dahouk et al., 2017	- [a]	- [a]	- [a]	MT637407	MT637414	MT637421
09RB8913	African bull frog (*Pyxicephalus edulis*)	Al Dahouk et al., 2017	- [a]	- [a]	- [a]	MT637408	MT637415	MT637422
10RB9205	African bull frog (*Pyxicephalus edulis*)	Al Dahouk et al., 2017	- [a]	- [a]	- [a]	MT637409	MT637416	MT637423
10RB9213	African bull frog (*Pyxicephalus edulis*)	Al Dahouk et al., 2017	- [a]	- [a]	- [a]	MT637410	MT637417	MT637424
10RB9215	African bull frog (*Pyxicephalus edulis*)	Al Dahouk et al., 2017	- [a]	- [a]	- [a]	MT637411	MT637418	MT637425
141012304	Bluespotted ribbontail ray (*Taeniura lymma*)	Eisenberg et al., 2016	- [b]	- [b]	- [b]	- [b]	- [b]	- [b]
B13-0095	Pac-Man frog (*Ceratophrys ornata*)	Soler-Llorens et al., 2016	- [b]	- [b]	- [b]	- [b]	- [b]	- [b]
UK8/14	Whites tree frog (*Litoria caerulea*)	Whatmore et al. 2015	- [a]	- [a]	- [a]			

[a] MLSA data retrieved from Brucella PubMLST database

[b] MLSA data retrieved from whole genome assemblies (GenBank assembly accession numbers: 141012304: GCA_900095155; B13-0095: GCA_001742815; NF2653: GCA_000177155)

Table S4.3. NCBI GenBank accession numbers for MLSA data and 16S sequences (where applicable) generated for field strains

Strain ID[(a)]	Identity[(b)]	*16S*	*gpd*	*dnaK*	*trpE*	*csdB*	*leuA*	*acnA*
CCM 7036 (D)	*O. intermedium*	-	MT637427	MT637477	MT637527	MT637577	MT637627	MT637677
CCUG 1047 (D)	*O. anthropi*	-	MT637428	MT637478	MT637528	MT637578	MT637628	MT637678
CCUG 12860 (D)	*O. anthropi*	-	MT637429	MT637479	MT637529	MT637579	MT637629	MT637679
CCUG 16508 (D)	*O. anthropi*	-	MT637430	MT637480	MT637530	MT637580	MT637630	MT637680
CCUG 17894 (D)	*O. anthropi*	-	MT637431	MT637481	MT637531	MT637581	MT637631	MT637681
CCUG 1821 (D)	*O. tritici*	-	MT637432	MT637482	MT637532	MT637582	MT637632	MT637682
CCUG 29689 (D)	*O. tritici*	-	MT637433	MT637483	MT637533	MT637583	MT637633	MT637683
CCUG 32009 (D)	*O. anthropi*	-	MT637434	MT637484	MT637534	MT637584	MT637634	MT637684
CCUG 33786 (D)	*O. anthropi*	-	MT637435	MT637485	MT637535	MT637585	MT637635	MT637685
CCUG 34735 (D)	*O. pseudintermedium*	-	MT637436	MT637486	MT637536	MT637586	MT637636	MT637686
CCUG 39736 (D)	*O. intermedium*	-	MT637437	MT637487	MT637537	MT637587	MT637637	MT637687
CCUG 43465 (D)	*O. pseudintermedium*	-	MT637438	MT637488	MT637538	MT637588	MT637638	MT637688
CCUG 43892 (D)	*O. pseudogrignonense*	-	MT637439	MT637489	MT637539	MT637589	MT637639	MT637689
CCUG 7349 (D)	*O. anthropi*	-	MT637440	MT637490	MT637540	MT637590	MT637640	MT637690
CLM26 (D)	*O. anthropi*	-	MT637441	MT637491	MT637541	MT637591	MT637641	MT637691
CLM6 (D)	*O. anthropi*	-	MT637442	MT637492	MT637542	MT637592	MT637642	MT637692
DSM 20150 (D)	*O. anthropi*	-	MT637443	MT637493	MT637543	MT637593	MT637643	MT637693

LMG 2134 (D)	*O. tritici*	-	MT637444	MT637494	MT637544	MT637594	MT637644	MT637694
LMG 2136 (D)	*O. anthropi*	-	MT637445	MT637495	MT637545	MT637595	MT637645	MT637695
LMG 3298 (D)	*O. anthropi*	-	MT637446	MT637496	MT637546	MT637596	MT637646	MT637696
LMG 3306 (D)	*O. intermedium*	-	MT637447	MT637497	MT637547	MT637597	MT637647	MT637697
LMG 35 (D)	*O. anthropi*	-	MT637448	MT637498	MT637548	MT637598	MT637648	MT637698
LMG379 (D)	*O. intermedium*	-	MT637449	MT637499	MT637549	MT637599	MT637649	MT637699
LMG 394 (D)	*O. anthropi*	-	MT637450	MT637500	MT637550	MT637600	MT637650	MT637700
LMG 401 (D)	*O. tritici*	-	MT637451	MT637501	MT637551	MT637601	MT637651	MT637701
LMG 5425 (D)	*O. intermedium*	-	MT637452	MT637502	MT637552	MT637602	MT637652	MT637702
LMG 5426 (D)	*O. intermedium*	-	MT637453	MT637503	MT637553	MT637603	MT637653	MT637703
LMG 5442 (D)	*O. anthropi*	-	MT637454	MT637504	MT637554	MT637604	MT637654	MT637704
LMG 5444 (D)	*O. anthropi*	-	MT637455	MT637505	MT637555	MT637605	MT637655	MT637705
LMG 5446 (D)	*O. intermedium*	-	MT637456	MT637506	MT637556	MT637606	MT637656	MT637706
RR (D)	*O. intermedium*	-	MT637457	MT637507	MT637557	MT637607	MT637657	MT637707
OgA9c (D)	*O. grignonense*	-	MT637458	MT637508	MT637558	MT637608	MT637658	MT637708
TA13 (D)	*O. intermedium*	-	MT637459	MT637509	MT637559	MT637609	MT637659	MT637709
TA93 (D)	*O. tritici*	-	MT637460	MT637510	MT637560	MT637610	MT637660	MT637710
TD30 (D)	*O. intermedium*	-	MT637461	MT637511	MT637561	MT637611	MT637661	MT637711
WS1830 (D)	*O. tritici*	-	MT637462	MT637512	MT637562	MT637612	MT637662	MT637712

WS1846 (D)	*O. oryzae*	-	MT637463	MT637513	MT637563	MT637613	MT637663	MT637713
R5/07 (UK)	*Pseudochrobactrum sp.*	MT625679	MT637464	MT637514	MT637564	MT637614	MT637664	MT637714
R16/08-3UT (UK)	*Pseudochrobactrum sp.*	MT625680	MT637465	MT637515	MT637565	MT637615	MT637665	MT637715
R17/08-1UD (UK)	*Pseudochrobactrum sp.*	MT625681	MT637466	MT637516	MT637566	MT637616	MT637666	MT637716
R22/05 (UK)	*Pseudochrobactrum sp.*	MT625682	MT637467	MT637517	MT637567	MT637617	MT637667	MT637717
R24/05 (UK)	*Pseudochrobactrum sp.*	MT625683	MT637468	MT637518	MT637568	MT637618	MT637668	MT637718
UK2/05 (UK)	*Pseudochrobactrum sp.*	MT625684	MT637469	MT637519	MT637569	MT637619	MT637669	MT637719
UK3/11-1 (UK)	*Paenochrobactrum sp.*	MT625685	MT637470	MT637520	MT637570	MT637620	MT637670	MT637720
UK5/07 (UK)	*Pseudochrobactrum sp.*	MT625686	MT637471	MT637521	MT637571	MT637621	MT637671	MT637721
UK14/09 (UK)	*Pseudochrobactrum sp.*	MT625687	MT637472	MT637522	MT637572	MT637622	MT637672	MT637722
UK20/09 (UK)	*Pseudochrobactrum sp.*	MT625688	MT637473	MT637523	MT637573	MT637623	MT637673	MT637723
UK34/07-2 (UK)	*Pseudochrobactrum sp.*	MT625689	MT637474	MT637524	MT637574	MT637624	MT637674	MT637724
VLA07/50 (UK)	*Ochrobactrum sp.*	MT625690	MT637475	MT637525	MT637575	MT637625	MT637675	MT637725
VLA10/07-E (UK)	*Pseudochrobactrum sp.*	MT625691	MT637476	MT637526	MT637576	MT637626	MT637676	MT637726

(a) Strains denoted (D) provided by Bundeswehr Institute of Microbiology (Munich, Germany); strains denoted (UK) obtained by the Animal and Plant Health Agency (Weybridge, UK)
(b) Identity based on published data (German strains: see Scholz et al., 2008b) or analysis of 16S rRNA sequence (UK strains)
